# The small world coefficient 4.8 ± 1 optimizes information processing in 2D neuronal networks

**DOI:** 10.1038/s41540-022-00215-y

**Published:** 2022-01-27

**Authors:** F. Aprile, V. Onesto, F. Gentile

**Affiliations:** 1 Department of Electric Engineering and Information Technology, University Federico II, 80125 Naples, Italy; 2 Institute of Nanotechnology, National Research Council (CNR‐NANOTEC), Campus Ecotekne, via Monteroni, Lecce, 73100 Italy; 3 Nanotechnology Research Center, Department of Experimental and Clinical Medicine, University of Magna Graecia, 88100 Catanzaro, Italy

**Keywords:** Systems biology, Biomedical engineering, Information technology

## Abstract

Small world networks have recently attracted much attention because of their unique properties. Mounting evidence suggests that communication is optimized in networks with a small world topology. However, despite the relevance of the argument, little is known about the effective enhancement of information in similar graphs. Here, we provide a quantitative estimate of the efficiency of small world networks. We used a model of the brain in which neurons are described as agents that integrate the signals from other neurons and generate an output that spreads in the system. We then used the Shannon Information Entropy to decode those signals and compute the information transported in the grid as a function of its small-world-ness (${\rm{SW}}$), of the length ($\triangle t$) and frequency ($f$) of the originating stimulus. In numerical simulations in which ${\rm{SW}}$ was varied between $0$ and $14$ we found that, for certain values of $\triangle t$ and $f$, communication is enhanced up to $30$ times compared to unstructured systems of the same size. Moreover, we found that the information processing capacity of a system steadily increases with ${\rm{SW}}$ until the value ${\rm{SW}}=4.8\pm 1$, independently on $\triangle t$ and $f$. After this threshold, the performance degrades with ${\rm{SW}}$ and there is no convenience in increasing indefinitely the number of active links in the system. Supported by the findings of the work and in analogy with the exergy in thermodynamics, we introduce the concept of exordic systems: a system is exordic if it is topologically biased to transmit information efficiently.

## Introduction

In biological systems, in tissues and organs, and the brain, the performance of a system depends less on the characteristics of a single cell and more on how those cells interact collectively to transport signals, information, or nutrients. The emergent properties of these systems arise from the cooperation of a great many elements and cannot be obtained or explained as the sum of the behaviors of each of their parts taken individually^1–6^.

Practically, we can gain access to the precious understanding of those systems by representing them as networks, in which the cells are the nodes and the interactions between cells are the links of the network. Since networks are measurable, one can then establish a relationship between the topology and the emergent properties of a system derived from the collective function of its many parts. Among the numerous variables that can describe the networks’ topology, the small-world coefficient has particular relevance because both experimental evidence and numerical simulations suggest that small-world systems can transport information more efficiently than periodic or random networks of the same size^7–12^. A network has a small-world topology if nodes of the network are separated from each other by a small number of steps, and very often small-world networks are characterized by a certain number of clusters with many node-node intracluster interactions and less intercluster connections^13–15^.

Nonetheless, while a variety of studies have examined how systems with a specific small-world topology behave under certain conditions, none of them illustrates how the information content of a system varies as a function of its small-world-ness. In this work, using numerical simulations we show how the efficiency of a 2-dimensional network of neurons changes as a function of its small-world characteristics.

To do so, firstly we generated a great many configurations ($\sim 1000$) with different topological characteristics. We placed in a fixed domain $500$ points randomly sampled from Gaussian distributions where the number, mean, and standard deviation of the distributions were varied over large intervals. Then, we connected the points of the distributions proportionally to the inverse of their distance and to the local density of other points in their neighbors. This wiring algorithm, developed by one of the authors of this work^16^, guarantees local and global connectivity, typical of small-world networks. By varying the parameters of the algorithm as described in the Methods of the work, we obtained networks with values of small-world-ness falling in the 0–14 interval.

The small-world-ness or small-world coefficient (${\rm{SW}}$) is a quantitative measure of the topological characteristics of a network relative to an equivalent random graph of that graph. It is defined in terms of the clustering coefficient (cc) and characteristic path length (cpl) as^17^:

1 $${\rm{SW}}=\frac{{{{\mathrm{cc}}}}_{{{\mathrm{graph}}}}/{{{\mathrm{cc}}}}_{{{\mathrm{rand}}}}}{{{{\mathrm{cpl}}}}_{{{\mathrm{graph}}}}/{{{\mathrm{cpl}}}}_{{{\mathrm{rand}}}}},$$

thus small-world networks have high clustering and short paths compared to random graphs of the same size. For a random graph, ${\rm{SW}}=1$.

**Fig. 1:**
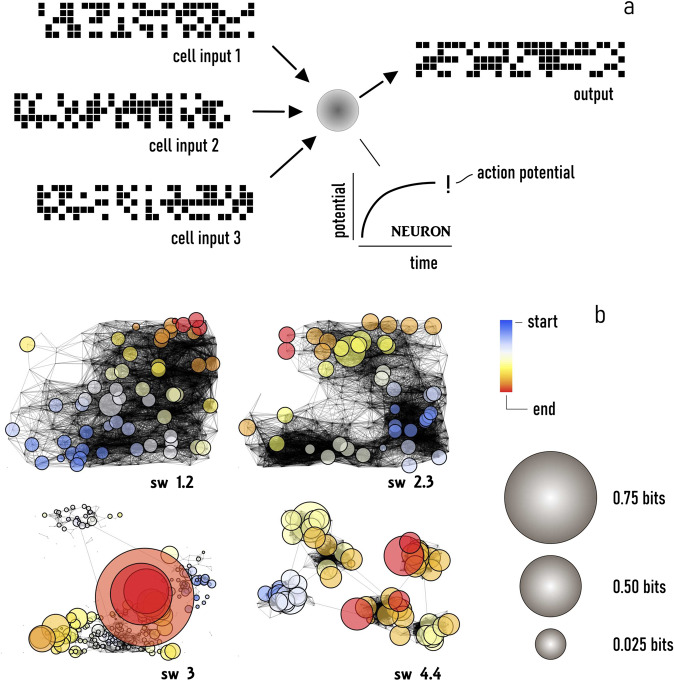
The neuronal network model. We simulated the transport of information in the networks, with their nodes represented as artificial neurons that integrate over time the current-pulse trains received as an input and produce, as an output, a discrete pattern of action potentials: in the model, the signals are represented as arrays of 0/1 (bits) (**a**). Information is decoded in the patterns of actions potentials: using information theory approaches and the Shannon information entropy, we obtained the information received and transmitted by each of the nodes of the networks (**b**).

After having generated configurations with different values of SW, we evaluated how a signal is transported in those networks where the elements of the networks are artificial neurons that receive as an input the signal from other neurons and pass it to the grid upon integration over space and time. This scheme is based on the repetition of a leaky integrate-and-fire model in the network as exhaustively reported in refs. ^18,19^. In analogy with the behavior of real neurons, the model generates as an output for each node of the grid a sequence of action potentials (train of spikes) that is encoded in the system as a binary sequence of $0$ and $1$ (Fig. 1a). Then, we used an information theory approach to decode the information stored in each node of the networks^20–23^. We computed for each sequence of $0$ and $1$ the associated value of Shannon Information Entropy $H$:

2 $$H=-\mathop{\sum }\limits_{i}^{S}P\,{\log }\left(P\right),$$

where the index $i$ runs over all the possible substates $s$ of the system, and $P$ is the probability of finding a specific substate $i$ in the originating sequence. In Eq. (2), the logarithm is taken in base $2$. Finally, we calculated for each node of the grid the information transported through that node as $I={H}_{0}-N$, where *H*_0_ is the entropy associated with an irregular, accidental stimulus, and $N$ is the entropy associated with a periodic, repetitive stimulus.

Using this scheme, we derived for each configuration the information transported in the networks as a function of time and position in the network (Fig. 1b). The values of information found for each node of the grid were then used to derive the information quantity, quality, and density over the entire network as a function of network topology (${\rm{SW}}$), and of the length $(\triangle t$) and frequency $(f$) of the originating stimulus.

## Results

### Measuring the performance of a network of neurons

**Fig. 2:**
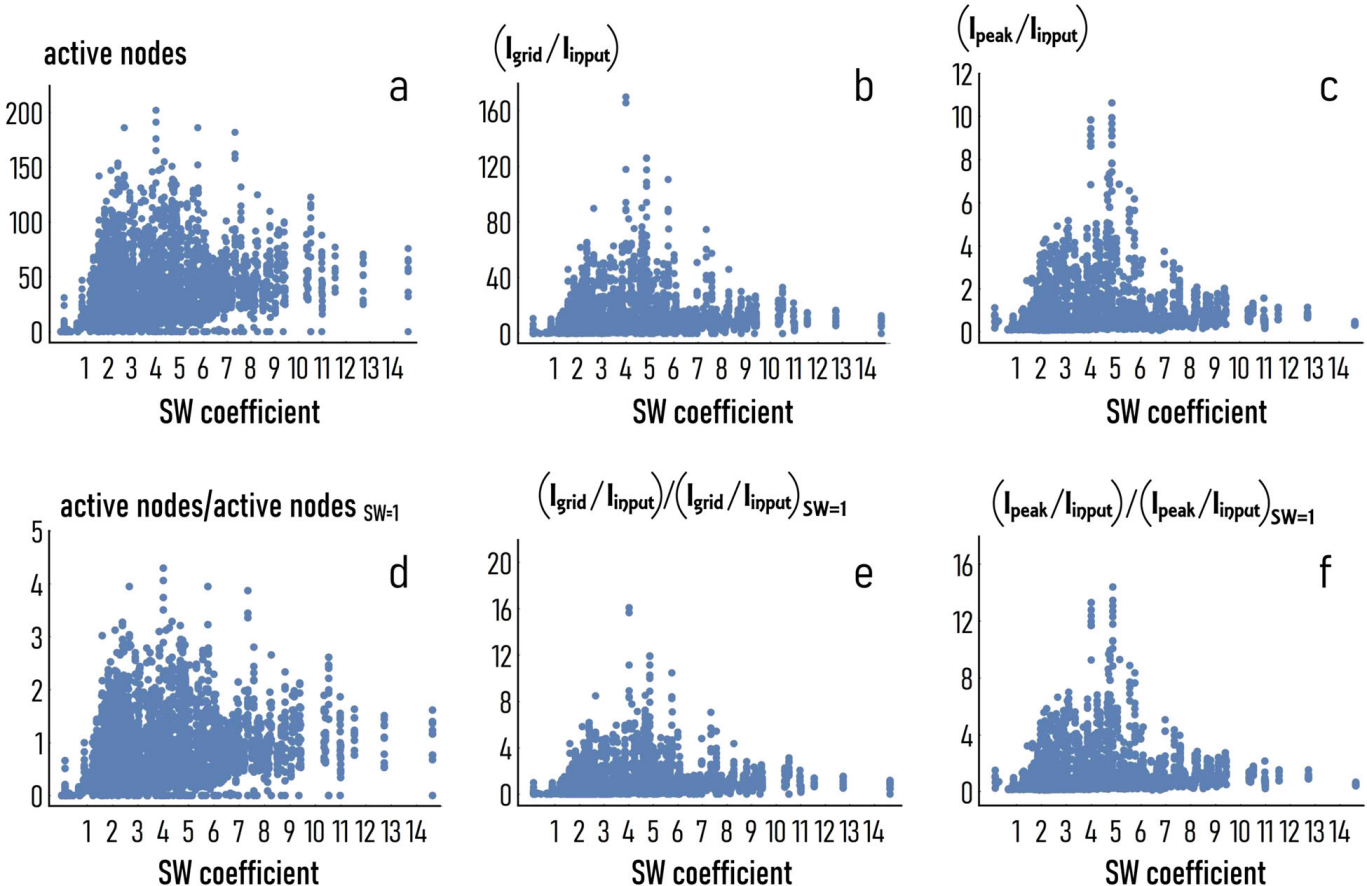
Applying repeatedly a random signal to nodes of the networks, we produced a disturbance that traveled in the system with a pace and amplitude that depended on the networks’ topology. To quantify the effect of small-world-ness on the information transported by the disturbance, we measured for each network topology (${\rm{SW}}$) the number of nodes reached by the signal (active nodes, **a**), and the total (${I}_{{{\mathrm{grid}}}}/{I}_{{{\mathrm{input}}}}$, **b**) and the peak information (${I}_{{{\mathrm{peak}}}}/{I}_{{{\mathrm{input}}}}$, **c**) in the network, compared to the same values associated to the initial stimulus. Then, we found how the number of active nodes (**d**), the normalized total (**e**), and peak information (**f**) behave relative to the values of these system’s variables determined for the special case ${\rm{SW}}=1$. The ratios reported in the insets **d**–**f** represent enhancement factors (${\eta }^{{{\mathrm{nodes}}}}$, ${\eta }^{{{\mathrm{grid}}}}$, ${\eta }^{{{\mathrm{peak}}}}$) and indicate how much the information flows in the network are enhanced due to the network topology. These diagrams were determined for a length and frequency of the initial stimulus of $\triangle t=72\,{{\mathrm{ms}}}$ and $f=133\,{{\mathrm{Hz}}}$.

We generated $\sim \!\!1000$ different networks of $500$ neurons in which the small-world-ness was varied between $0$ and $14$ as explained in the methods of the paper. In Supplementary Fig. 1a, we show examples of configurations that we obtained with this method. For these, the ${\rm{SW}}$ value ranges from $1.3$ to $4.4$: in this interval the associated graphs transition from uniform (${\rm{SW}}=1.3$) to highly clustered layouts, with elements of the graph compartmentalized in a few clusters with many links per cluster (${\rm{SW}}=4.4$). In Supplementary Fig. 1b, we report the total number and distribution of configurations that we have generated for this study. To test the networks, we analyzed how a stimulus applied to a randomly selected node propagates in the system. For this configuration, the input signal was a sequence of $3\times 8$ letters, i.e., random variables that can take only the values $0$ or $1$. Since each letter represents a duration of $\delta t=3\,{{\mathrm{ms}}}$, the entire input signal has a length of $\triangle t=72\,{{\mathrm{ms}}}$. Moreover, the probability for a letter of being $1$ was fixed as $p=0.4$, implying that the sampling frequency of the initial stimulus is $f=p/\delta t \sim 133\,{{\mathrm{Hz}}}$. To increase confidence in the results, we simulated the propagation of a signal $10$ different times for each network topology, thus the total number of simulations is $n=\mathrm{10,000}$ for a fixed $\triangle t$ and $f$. We evaluated the performance of the networks using $3$ different metrics, i.e., the number of nodes in the network through which the signal propagates (active nodes), the information transported all over the nodes of the grid scaled to the value of information contained in the stimulus (*I*_grid_/*I*_input_), the maximum information transported in the grid divided by the information of the stimulus (*I*_peak_/*I*_input_). Figure 2a–c are scatter plots of the measured number of active nodes, of *I*_grid_/*I*_input_ and *I*_peak_/*I*_input_ against the small-world coefficient. We observe that the number of active nodes (${\rm{AN}}$) steadily increases for increasing values of ${\rm{SW}}$ between ${\rm{SW}}=0$ and ${\rm{SW}}=4$, in this range ${\rm{AN}}$ transitions from ${\rm{AN}}=0$ to ${\rm{AN}}=200$, then, the maximum value of ${\rm{AN}}$ decreases with ${\rm{SW}}$, being ${\rm{AN}} \sim 150$ for ${\rm{SW}} \sim 5$, ${\rm{AN}} \sim 180$ for ${\rm{SW}} \sim 6$ and ${\rm{SW}} \sim 7$, ${\rm{AN}} \sim 100$ for ${\rm{SW}} \sim 11$, ${\rm{AN}} \sim 70$ for ${\rm{SW}} \sim 13$ (Fig. 2a). A similar trend is observed for *I*_grid_/*I*_input_ (Fig. 2b) and *I*_peak_/*I*_input_ (Fig. 2c), for which the total and peak values of information increase steeply with ${\rm{SW}}$ and reach a maximum at an optimal value of small-world-ness that is estimated as ${{\rm{SW}}}^{o} \sim 4$ for *I*_grid_/*I*_input_ and ${{\rm{SW}}}^{{\mathrm{o}}} \sim 5$ for *I*_peak_/*I*_input_. For these values of small-world-ness, the total information transported in the network and the peak information are $\sim \!\!160$ times and $\sim \!\!11$ times higher, respectively, than the information contained in the stimulus.

Since we are interested in understanding to which extent network topology affects information, it is relevant determining how ${\rm{AN}}$, *I*_grid_/*I*_input_, *I*_peak_/*I*_input_ compare to the values of these variables found for ${\rm{SW}}=1$. The non-dimensional variables ${\eta }^{{{\mathrm{nodes}}}}={\rm{AN}}/{{\rm{AN}}}^{{\rm{sw}}=1}$, ${\eta }^{{{\mathrm{grid}}}}={I}_{{{\mathrm{grid}}}}/{I}_{{{\mathrm{grid}}}}^{{{\mathrm{sw}}}=1}$, ${\eta }^{{{\mathrm{peak}}}}={I}_{{{\mathrm{peak}}}}/{I}_{{{\mathrm{peak}}}}^{{{\mathrm{sw}}}=1}$, indicate the enhancement of the active nodes, of the total and peak information in a grid because of the grid topology described by the SW coefficient. The scatter plot in Fig. 2d illustrates that the total number of active nodes is up to $4$ times higher in a topologically biased network than in a random network of the same size. In the same way, Fig. 2e, f indicates that—for these values of the model parameters—the total information transported in a grid may be up to $16$ times higher, and the peak information up to $14$ times higher, compared to the corresponding values measured in random unstructured graphs.

### Effect of signal length

**Fig. 3:**
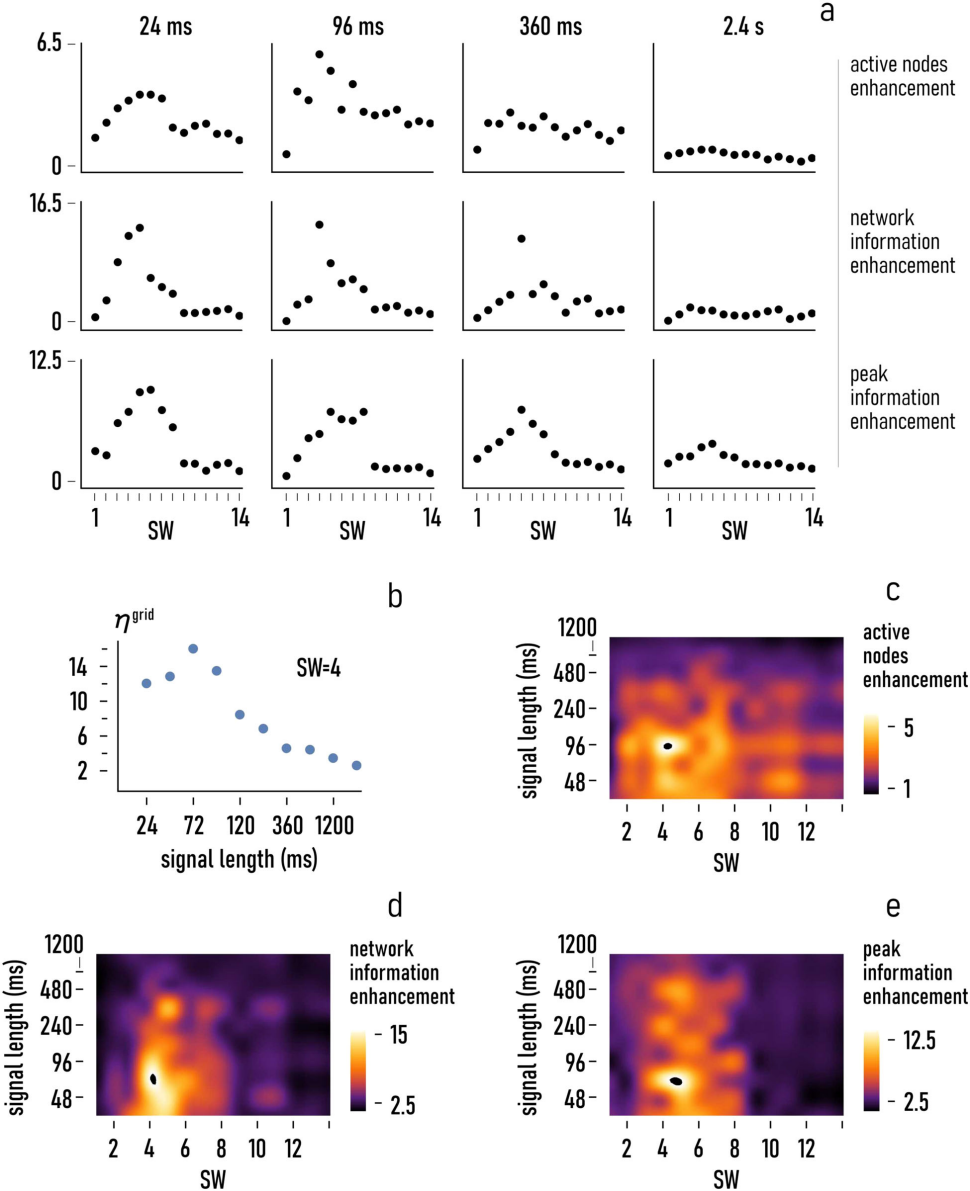
Effect of signal length. Varying the length of the initial stimulus (Δ*t*) in discrete intervals, we observed the efficiency of the networks (*η*^nodes^, *η*^grid^, *η*^peak^) exhibits different sensitivities to the small-world-coefficient (**a**). Specifically, varying for a fixed ${\rm{SW}}=4$ $\triangle t$ in the 24–2400 ms interval, we observe that ${\eta }^{{{\mathrm{grid}}}}$ ranges from about $2$ at $\triangle t=2400\,{{\mathrm{ms}}}$ to about $12$ for $\triangle t=72\,{{\mathrm{ms}}}$ (**b**). Density plots of the network enhancement factors ${\eta }^{{{\mathrm{nodes}}}}$ (**c**), ${\eta }^{{{\mathrm{grid}}}}$ (**d**), ${\eta }^{{{\mathrm{peak}}}}$ (**e**). In deriving the plots, we fixed the value of frequency of the originating distubance as $f=133\,{{\mathrm{Hz}}}$.

The values of active nodes, total information, and peak information efficiency were determined in the previous section for a fixed value of length of the originating signal, i.e., $\triangle t=72\,{{\mathrm{ms}}}$. We have therefore performed a test campaign where we varied the signal length in the $\triangle t=24-2400\,{{\mathrm{ms}}}$ range to examine how information is affected by the initial stimulus. In the tests, the sampling frequency of the input signal was hold fixed as $f \sim 133\,{{\mathrm{Hz}}}$. Figure 3a reports the active nodes (*η*^nodes^), the total information (*η*^grid^) and the peak information (*η*^peak^) efficiency of the network as function of the small-world-ness for different values of $\triangle t$, i.e., 24 ms, 96 ms, 360 ms, and 2.4 s. Diagrams in the figure show that the smaller the duration of the stimulus, the higher the values of efficiency associated to the metrics used in this study. In Fig. 3b, we report the total information enhancement factor, *η*^grid^, as a function of $\triangle t$ for a fixed value of small-world-ness ${\rm{SW}}=4$. For this specific configuration, the efficiency in the grid varies from ${\eta }^{{{\mathrm{grid}}}} \sim 12$ for $\triangle t=24\,{{\mathrm{ms}}}$, to ${\eta }^{{{\mathrm{grid}}}} \sim 15$ for $\triangle t=72\,{{\mathrm{ms}}}$, to ${\eta }^{{{\mathrm{grid}}}} \sim 2$ for $\triangle t=2400\,{{\mathrm{ms}}}$. Thus, excluding the low-millisecond range, the efficiency in the grid decays with $\triangle t$ with a total variation of $\sim 10$ points of efficiency over two decades of $\triangle t$. To visualize at a glance the trend of the enhancements—of active nodes, total information, and peak information—we report in Fig. 3c–e, *η*^nodes^, *η*^grid^, and *η*^peak^ as a function of the characteristic ${\rm{SW}}$ and $\triangle t$ of a network. In the plots, ${\rm{SW}}$ and $\triangle t$ were varied in the 0–14 and the 24–2400 ms range, respectively. The density plots in the figure indicate that the active nodes increment (*η*^nodes^) exhibits a moderate sensitivity to ${\rm{SW}}$ and $\triangle t$, with high values of enhancement near five within a large space of the variables, with ${\rm{SW}}$ in the 2–8 interval and $\triangle t$ comprised between $24$ and ~480 ms (Fig. 3c). In contrast, *η*^grid^ and *η*^peak^ show a higher sensitivity to ${\rm{SW}}$ and $\triangle t$. The largest values of efficiency are measured for values of small-world-ness greater than $\sim \!\!2.5$ and smaller than $\sim \!\!6$, and values of signal length generally lower than ~100 ms. In this interval, the maximum values of efficiency are found as ${\eta }^{{{\mathrm{grid}}}} \sim 16$ and ${\eta }^{{{\mathrm{peak}}}} \sim 14$ (Fig. 3d, e).

### Effect of signal frequency

**Fig. 4:**
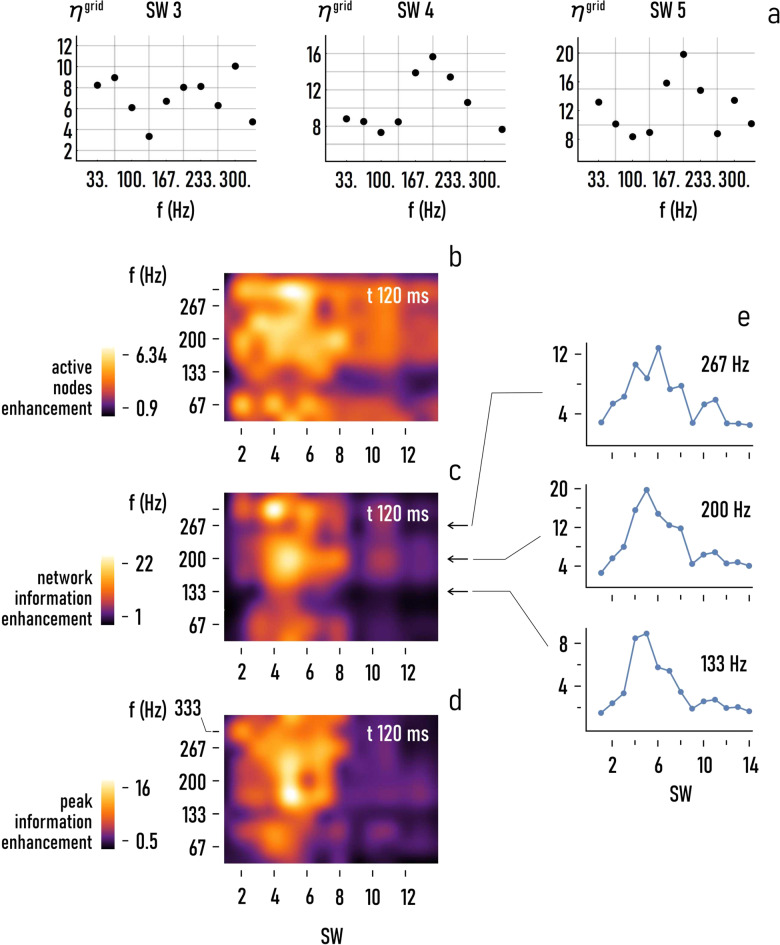
Effect of signal frequency. As with Δ*t*, for constant values of SW the efficiency of the grid *η*^grid^ varies for varying values of the frequency of the originating input *f* (**a**). The density plots of ${\eta }^{{{\mathrm{nodes}}}}$ (**b**), ${\eta }^{{{\mathrm{grid}}}}$ (**c**), ${\eta }^{{{\mathrm{peak}}}}$ (**d**) as a function of ${\rm{SW}}$ and $f$ show the combined influence of topology and frequency on the performance of the grid ($\triangle t=120\,{{\mathrm{ms}}}$). The diagrams of ${\eta }^{{{\mathrm{grid}}}}$ against ${\rm{SW}}$ extracted from the density plot in **c** for the values of frequency $f=133$, $\,200$, $267\,{{\mathrm{Hz}}}$, and $\triangle t=120\,{{\mathrm{ms}}}$ (**e**).

We then examined how the frequency of the input signal influences the performance of the system. To this end, we launched a series of simulations where, for a fixed signal length $\triangle t=120\,{{\mathrm{ms}}}$, the frequency $f$ of the initial disturbance was varied between *f* ~ 33 Hz and *f* ~ 333 Hz. Figure 4a reports the total information efficiency (*η*^grid^) as a function of $f$ for fixed values of small-world-ness: sw = 3, 4, 5. For these values of the model parameters, *η*^grid^ oscillates between a minimum and a maximum value of efficiency with the maximum occurring for frequencies generally larger than 200 Hz. This trend is mostly maintained for the three metrics used in this study. The density plots of *η*^nodes^, *η*^grid^, and *η*^peak^ as a function of ${\rm{sw}}$ and $f$ in Fig. 4b–d show that the networks optimize their performance for small-world coefficient comprised between $\sim \!\!2$ and $\sim \!\!8$, and for values of frequency larger than ~133 Hz. In this range, the maximum enhancement factor is $\sim \!\!6.34$ for the active nodes of the graph, $\sim \!\!22$ for the total information transported in the grids, $\sim \!\!16$ for the peak information. The diagram of *η*^grid^ as a function of ${\rm{sw}}$ for fixed values of frequency—$f=133,\,200,\,267\,{{\mathrm{Hz}}}$ (Fig. 4e), shows at any rate that the systems seem to exhibit a higher sensitivity to ${\rm{SW}}$ than to $f$. The maximum change of efficiency per change of ${\rm{sw}}$ is ${{s}} \sim 18/14 \sim 1.29$, calculated for a central frequency of *f* = 200 Hz over the 1–14 SW range. In contrast, the maximum change of efficiency per change of frequency in the considered range of values is ${{s}} \sim 10/70 \sim 0.15\,/{\rm{Hz}}$, calculated for ${\rm{SW}}=5$ in the 133–200 Hz interval (Fig. 4e).

All the enhancement factors of active nodes (*η*^nodes^), total information (*η*^grid^), and peak information (*η*^peak^) transported in the grids as a function of the small-world-ness of the networks of neuronal cells, for different values of length and frequency of the input signal used in this study are reported in the separate Supplementary Information section 2.

Furthermore, we have determined the performance of small-world networks in the 1–14 SW spectrum range quantitatively. To do this, we have introduced another parameter, the quality factor, that quantifies how the efficiency of a network, the peak efficiency, and the active nodes enhancement factor vary as a function of ${\rm{SW}}$. The quality factor, ${Q}^{i-f}$, is the efficiency increment expressed in terms of percentage, evaluated between an initial ($i$) and final ($f$) value of small-world-ness. Thus, to make an example, ${Q}^{1-4}=\left({\eta }_{4}-{\eta }_{1}\right)/{\eta }_{1}$. The quality factor is non-dimensional, similar to the efficiency. In the separate Supplementary Information section 3, we report the values of quality factor associated to *η*^grid^, *η*^peak^, and *η*^nodes^ calculated for different combinations of signal length ($\triangle t$) and signal frequency ($f$), and for values of the small world coefficient spanning the 1–14 interval. Overall, the results of the analysis are consistent with previously reported findings. The maximum increment of efficiency is found for intermediate values of small-world ness (${\rm{SW}}=4-5$), starting from an initial layout with either low (1) or high (14) SW coefficients. For certain combinations of the driving frequency and parameters of the model, the quality factors reach values as high as $\sim \!\!1250 \%$ (${Q}_{{{\mathrm{grid}}}}^{1-4}$), $\sim \!\!1000 \%$ (${Q}_{{{\mathrm{peak}}}}^{1-5}$), and $\sim \!\!320 \%$ (${Q}_{{{\mathrm{nodes}}}}^{1-5}$).

### The value of small-world-ness that optimizes performance of the networks

**Fig. 5:**
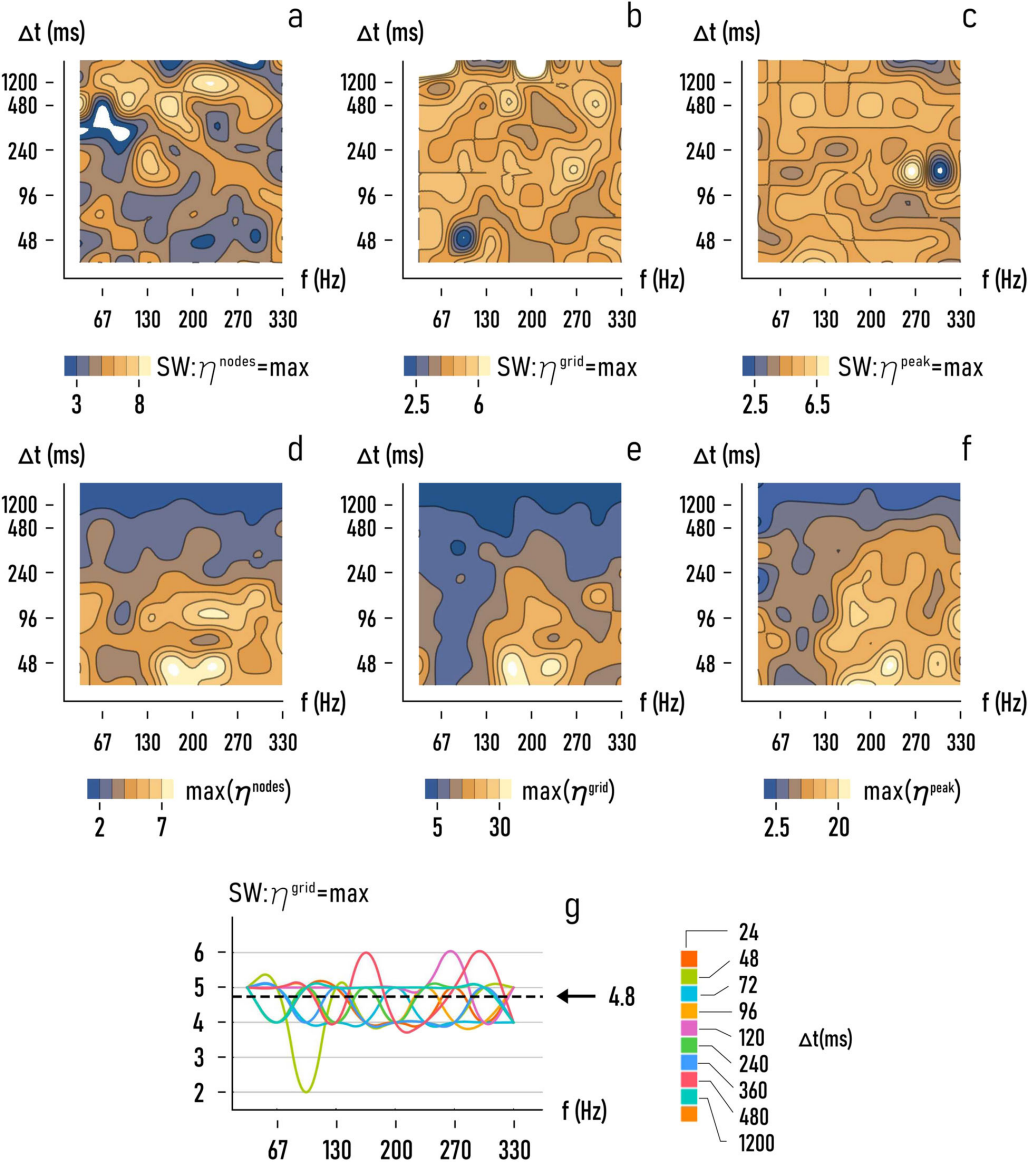
Optimal small-world-ness of the networks. For different combinations of $f$ and $\triangle t$, we determined the optimal values of small-world-ness ${\rm{SW}}$ for which *η*^nodes^ (**a**), *η*^grid^ (**b**) and *η*^peak^ (**c**) are maximum in the networks, and the corresponding maxima (**d**–**f**). For the particular case of *η*^grid^, we observe that optimal small-world-ness assuring the maximum efficiency in the grid oscillates around the mean value ${\rm{SW}}=4.8\pm 1$ (**g**). The white regions in the diagrams indicate values that are out-of-range, i.e., values of the functions that fluctuate either below or above the minimum (maximum) of the plot range identified through the color legend bar that accompany each diagram. These areas are ascribable to interpolation errors in the graphical representation of the values as smooth functions of the variables and do not affect to any extent the findings and conclusions of the study.

**Fig. 6:**
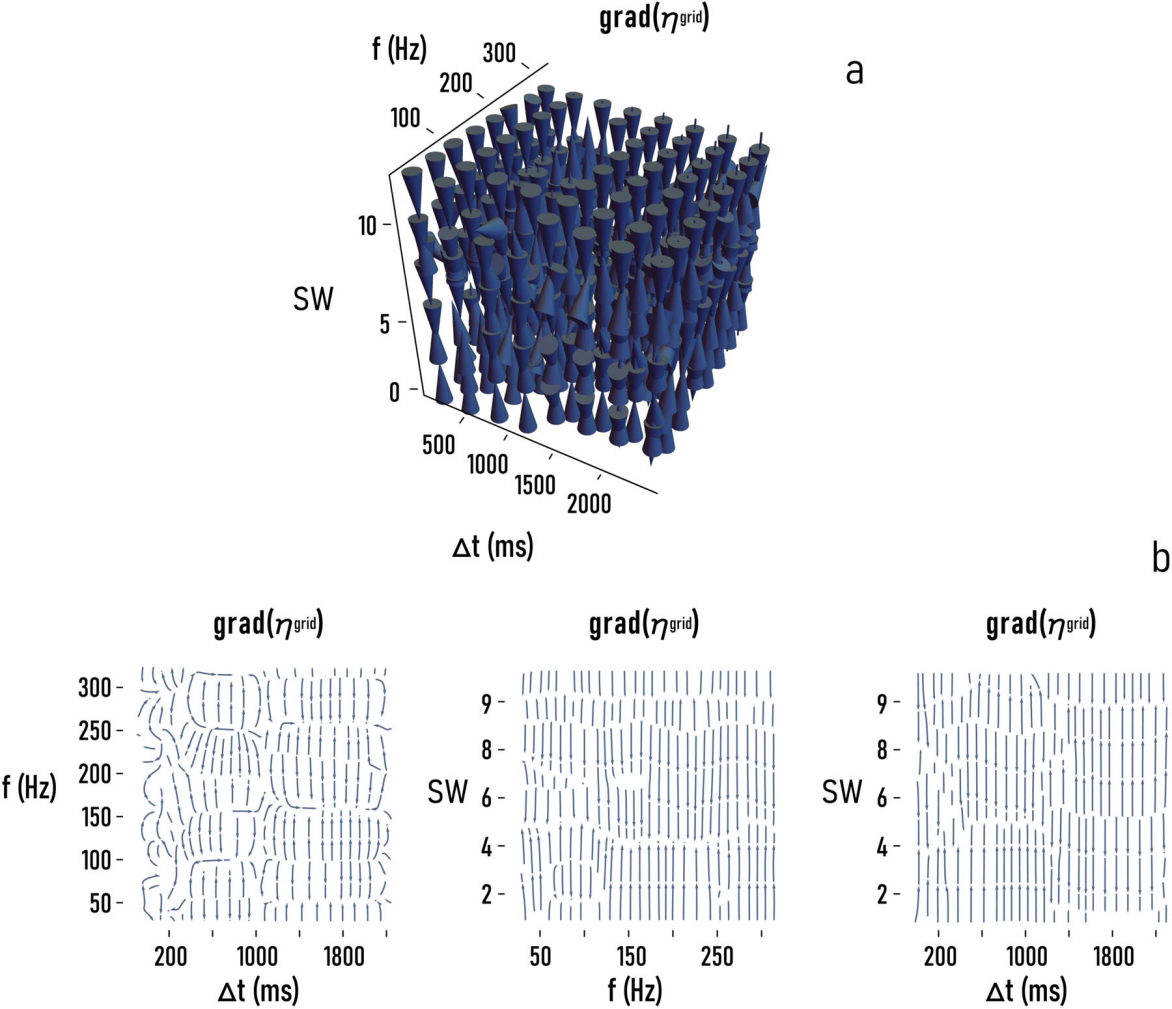
The enhancement factor of total information in the grid *η*^grid^ is a function of the model parameters: Δ*t*, *f*, and SW. To show that *η*^grid^ exhibits a greater sensitivity to ${\rm{SW}}$ than to $\triangle t$ or $f$, we report in **a** the gradient of *η*^grid^ $\left(\nabla {\eta }^{{{\mathrm{grid}}}}\right)$ as a function of $\triangle t$, $f$, and ${\rm{SW}}$, and in **b** $\nabla {\eta }^{{{\mathrm{grid}}}}$ as a function of those variables taken two at the time.

Results of the simulations indicate that the number of active nodes, the total information transported in a network, and the peak of information depend significantly on the topology of the network and are influenced more weakly by the length $\triangle t$ and the frequency $f$ of the input signal. For each of the $\triangle t$ and $f$ used in the work, it is relevant to determine the value of small-world-ness at which the efficiency of the network hits a maximum, and the magnitude of the maximum. Figure 5a–c reports as a function of $f$ and $\triangle t$ the values of ${\rm{SW}}$ that maximize *η*^nodes^, *η*^grid^, and *η*^peak^ in the networks. For these values of ${\rm{SW}}$, Fig. 5d–f shows the corresponding optimal values of *η*^nodes^, *η*^grid^, and *η*^peak^. Diagrams show that the best values of small-world-ness fall in narrow intervals: 3–8 for *η*^nodes^, 2.5–6 for *η*^grid^, 2.5–6.5 for *η*^peak^. In these intervals, the number of active nodes, and the total and peak information transported in a grid, may be enhanced up to $\sim \!\!7$, $\sim \!\!30$, or $\sim \!\!20$ fold, respectively, because of the network topology. Notably, these intervals may be still narrower if some fluctuations are removed from the system’s response. Consider as an illustration the enhancement factor—*η*^grid^. For this variable, we report in Fig. 5g the optimal small-world-ness (sw_o_) for which the efficiency is maximized as a function of frequency, for different values of the signal length. Much of the response of the system is comprised between $4$ and $6$, with oscillations below $4$ only limited to $\triangle t=48\,{{\mathrm{ms}}}$ and in any case restricted to values of frequency lower than 130 Hz. Also considering these variations, the mean value of sw_o_ is $\sim \!\!4.8\pm 1$, i.e., the optimal value of small-world-ness for which a system optimizes information flows oscillates weakly around the mean, suggesting that the optimal topology of a network is only moderately influenced from $f$ and $\triangle t$. Simulations over a wide variety of configurations and conducted for a broad spectrum of $f$ and $\triangle t$ suggest that there exists a specific topology for which information is maximized, independently from $f$ and $\triangle t$—and this topology is described by the sole parameter ${\rm{SW}} \sim \!\!4.8\pm 1$. The fact that information increments depend less on $f$ and $\triangle t$ and more on how neurons are organized (${\rm{SW}}$) is confirmed by the diagrams in Fig. 6.

Part a of Fig. 6 is a 3d vector plot from the gradient *η*^grid^ of the enhancement factor of information calculated with respect to the length ($\triangle t$) and frequency ($f$) of the traveling disturbance, and of the small-world-coefficient of the networks—${\rm{SW}}$. Thus, the direction of the cones indicates the lines along which the rate of change of *η*^grid^ is maximum, while the magnitude of the cones is proportional to the intensity of the rate of change. In the same way, the vector field plot in Fig. 6b indicates the gradient of *η*^grid^ with respect to the variables of the problem taken two at the time (i.e., (i) $f$ and $\triangle t$ for a fixed ${\rm{SW}}=4$, (ii) $f$ and ${\rm{SW}}$ for a fixed $\triangle t=48\,{{\mathrm{ms}}}$, (iii) $\triangle t$ and ${\rm{SW}}$ for a fixed $f=100\,{{\mathrm{Hz}}}$). The streamlines in Fig. 6b show the local direction of the vector field at every point.

In any case, the patterns of maximum variation of *η*^grid^ are aligned with ${\rm{SW}}$, and correlate very feebly with $f$ and $\triangle t$.

## Discussion

In several tests in which the small-world-ness $({\rm{SW}})$, and the signal length $(\triangle t)$ and frequency $(f)$ were varied over large intervals, we found that $I$ shows a very high sensitivity to ${\rm{SW}}$, and a less relevant sensitivity to $\triangle t$ and $f$. Moreover, we found that the optimal value of small-world-ness for which $I$ is maximized is ${\rm{SW}}=4.8\pm 1$. For this value of ${\rm{SW}}$, the total information transported in the grid is more than $30$ times larger than the information processed in an equivalent random network of the same size. Moving away from this value of topology, either downward or upward, the ability of the grid to process information becomes sub-optimal.

The observation that the increment of information in a network is mostly imputable to the small-world coefficient is notable: it suggests that the information gained from a system can be predicted from its topology. Diagrams that map the information efficiency of a network to its value of small-world-ness, as those derived in this work, can serve as a preliminary reference for the design and development of complex systems, biological systems, and the bio-interface for applications in tissue engineering, neuromorphic engineering, regenerative medicine, the study of neurodegenerative diseases.

These design maps indicate the maximum useful information that can be processed by a system due to its shape. In analogy with exergy in thermodynamics, we propose to designate this maximum information with the term exorder information: it is the maximum available information in a system as determined by the order or structure of its components (the exergy in thermodynamics indicates the maximum available work obtainable from a system). Thus, a system of many elements is exordic if it assumes a configuration that guarantees maximum possible information flows.

The hypothesis that we put forward in the paper and that we proved using numerical simulations, is that the exorder information condition is met for values of small-world-ness falling in the narrow interval $4.8\pm 1$. This is substantiated by the results of the work and especially by the diagram shown in Fig. 5g, where the value of the small-world coefficient for which the performance of a system is optimized oscillates around $4.8$. Thus, a system is exordic if it is topologically biased to transmit information efficiently; moreover, results of the paper indicate that bi-dimensional systems are exordic for a value of small-world-ness of about $4.8$. This has some consequences.

Firstly, the performance of complex systems is related to the sole small-world coefficient. Irrespective of the frequency and length of the originating signal, periodic, disordered, or regular networks are equivalent from the information perspective if they have the same ${\rm{SW}}$ value. Thus, the scientist or biomedical engineer in the process of designing a system, should arrange the components of that system in a fashion to ensure intermediate values of ${\rm{SW}}$ close to $5$—if he wants to maximize signaling. This is especially relevant in neuromorphic engineering, where the architecture of computing devices is modeled after the shape of true biological neuronal networks, such as the brain. In this case, one can achieve maximum computation speed, efficiency, and power by pursuing the ${\rm{SW}} \sim 5$ rule.

Further to this end, the results of the paper reinforce the view that the topology of a system and the information transported within that system are equivalent. The equivalence principle, suggested in previous reports^24–26^, states that the information and the topology of a system are of a similar nature—with the former heavily influenced by the latter.

Among the many several possible applications of this criterion, we may enumerate the study of neurodegenerative diseases and neural tissue repair and regeneration. In one and the other case, one can estimate the behavior and status of the system (i.e., neuronal tissue, regions of the brain or of the nervous system) from its shape without the burden of recording the activity from numerous neurons with single-cell resolution, identify the location of recorded neurons, or detect non-active neurons during the observation period. For these reasons, in perspective the concept devised and developed in this study can be used as an instrument in neurology and used in concert to other assessed techniques, such as nMRI, fMCI, or single-cell recording, to decode the intimate nature of the brain.

However, despite the far-reaching consequences of the results, the extent of validity of the model has to be discussed. The small world coefficient $4.8$ that we found to optimize the network performance is based on some assumptions.

The first and foremost is that the networks that we have examined are 2d. This simplification enabled to examine a great many of network topologies and configurations, but at the same time inevitably limits the field of applicability of the results to two-dimensional systems, such as 2d cell cultures; and if results are instead applied to 3d systems, such as cellular spheroids, neuroblastoma in vitro models, or other similar systems, this is done at the cost of, at best, an approximation.

The dependence of the results on the model used to generate random graphs is, to the opinion of the Authors, less a limitation of the model and more linked to the very definition of small-world-ness (see the “Methods” of the article for further details). The $4.8$ figure that emerged from the simulations is tightly connected to the fact that the networks used as a reference to measure the topology of our structures are random, uniform Erdos–Renyi graphs. This lies at the basis of the notion of small-world coefficient, for how it has been conceptualized by Humphries^17^ and other scholars who first studied the argument in detail.

In the last place, the results of the study rely on the generalized version of the leaky integrate and fire (LIF) model and the differential Eq. (6), which is described in the “Methods” of the article. The equation and the voltage discharged by the neurons depend on the model parameters *C*_m_ and *g*_l_, i.e., the capacitance and conductance of the cell membrane, and on *V*_o_, that is the potential at the rest of the system. Other parameters that influence the solution are the time constant $\tau$ and *I*_stim_, which in turn depends on how a neuron is connected to other active units of the system and ultimately to the topology of the network. In our study, we have used the following values for the model parameters: ${C}_{{\mathrm{m}}}=300\,{{\mathrm{pF}}}$, ${g}_{{\mathrm{l}}}=0.1\,\mu {\mathrm{S}}$, $\tau =3\,{{\mathrm{ms}}}$, ${V}_{{\mathrm{o}}}=6\,{{\mathrm{mV}}}$, adopted from previously reported studies^18,19^. While it is possible that the optimal value of small-world-ness of the networks may vary by changing the constant of the LIF model—and future studies are necessary to investigate further this aspect—nonetheless it is the opinion of the authors that the results of this study are robust to changes of the variables of the neural model. The values assigned to *C*_m_, *g*_l_, $\tau$, and *V*_o_, modulate the time-response of a neuron through a differential equation: thus, in the final instance, they influence the pattern of action potentials (signals) that are generated by a neuron and passed to other units in the net. In numerical simulations (as for some examples, those reported in Figs. 3–6) we have shown that the information quantity, quality, and density transported in the systems show a very low sensitivity to the length and frequency of the traveling disturbance. Supported by these results and in the same way, we suppose that the enhancement of information in the grids is only marginally influenced by the parameters of the LIF models, and that the small-world coefficient of $4.8$ that emerged from this study maintains the character of generality.

## Methods

### Generating small-world networks

To examine how signals propagate in topologically biased networks, we generated $\sim \!\!1000$ configurations of $500$ nodes with values of small-world-ness varying between $0$ and $14$. Firstly, we sampled $500$ points in a plane from Gaussian distributions of the type:

3 $$g\left(x,y\right)=\frac{1}{\sigma \sqrt{2\pi }}{e}^{-\frac{{\left({\left(x-{x}_{{\mathrm{o}}}\right)}^{2}+{\left(y-{y}_{{\mathrm{o}}}\right)}^{2}\right)}^{0.5}}{2{\sigma }^{2}}},$$

where $x$ and $y$ designate the spatial coordinates, $o=\left({x}_{{\mathrm{o}}},{y}_{{\mathrm{o}}}\right)$ is the center of the distribution, and ${\sigma }^{2}$ is its variance. To generate the configurations, we sampled points from $6$ different distributions with centers randomly picked in a $10\times 10$ interval, and values of standard deviations oscillating around the central value $\sigma =0.2$ with an amplitude $\triangle \sigma =0.1$. Then, we routed the nodes using an algorithm developed by us^16^. The code makes a decision on whether two points $i$ and $j$ are connected based on a mixed rule that combines two wiring probabilities.

The first probability (distance rule) is proportionate to the inverse of the distance between $i$ and $j$: ${p}_{1}^{{ij}}=\alpha\,{\exp }\left(-\beta {d}_{{ij}}/l\right)$, where $l$ is the maximal inter-nodal distance and $\alpha$ and $\beta$ are model parameters. This rule generates short-range connections and is a variation of the celebrated Waxman algorithm^27^. According to this rule, a link between $i$ and $j$ is established if ${p}_{1}^{{ij}}\, > \,{P}_{{\mathrm{w}}}$, where *P*_w_ is a constant.

The second probability (density rule) evaluates whether both $i$ and $j$ have high local density and relatively high distance from other nodes with higher density. Inspired by the work of Rodrigueze and Laio^28^, who developed an algorithm that spots cluster centers even in distributions without spherical symmetry, we associated to each node of the dataset ($k$) the number ${p}_{2}(k)={\exp }\left(-\left({\Gamma }_{{\max }}/\Gamma -1\right)\right)$, where $\Gamma =\rho {d}_{{\min }}$. In this identity, $\rho$ is the local density of $k$, determined as the number of points falling within a cutoff distance ${\delta }_{{{\mathrm{co}}}}$ from $k$; ${d}_{{\min }}$ is the minimum distance to other points with higher density than $k$; ${\Gamma }_{{\max }}$ is the maximum $\Gamma$ over all the $k$. Thus, the larger ${p}_{2}(k)$, the higher the probability of $k$ being a cluster center, with high local distance and relatively high distance from other cluster centers. With these premises, the condition of existence of a link between each node pairs is defined as ${p}_{2}^{i}\, > \,{P}_{{\mathrm{d}}}\wedge {p}_{2}^{j}\, > \,{P}_{{\mathrm{d}}}$, where *P*_d_ is a constant. This rule generates long-range interactions.

The mixed rule combines the distance and the density rule to determine whether nodes $i$ and $j$ are connected as ${{{\mathrm{link}}}}^{{ij}}={p}_{1}^{{ij}}\, > \,{P}_{{\mathrm{w}}}\vee \left({p}_{2}^{i}\, > \,{P}_{{\mathrm{d}}}\wedge {p}_{2}^{j}\, > \,{P}_{{\mathrm{d}}}\right)$. Thus, two nodes are connected if they pass the distance *or* the density rule test. To generate the configurations used in this study, we varied the values of the model parameters in the following intervals: ${P}_{{\mathrm{w}}}=0.90-1.0$, ${P}_{{\mathrm{d}}}=0.90-1.0$, $\beta =0.2-0.8$, ${\delta }_{{{\mathrm{co}}}}=0\pm 0.4$, and fixed $\alpha$ as $\alpha =1$.

### Network analysis

The topological characteristics of the networks, such as the adjacency matrix, containing the information about the connectivity among node pairs in the network, the mean clustering coefficient, and the characteristic path length of the networks, were determined using the methods reported in a separate Supplementary Information section 4.

### Measuring the small world coefficient of the networks

We measured the small-world coefficient of the graphs using the formula^17^:

4 $${\rm{SW}}={\rm{\gamma }}/{\rm{\varphi }},$$

with

5 $${\rm{\gamma }}={{\mathrm{Cc}}}_{{{\mathrm{graph}}}}/{{\mathrm{Cc}}}_{{{\mathrm{rand}}}},\,\,{\rm{\varphi }}={{\mathrm{Cpl}}}_{{{\mathrm{graph}}}}/{{\mathrm{Cpl}}}_{{{\mathrm{rand}}}}.$$

${{\mathrm{Cc}}}_{{{\mathrm{graph}}}}$ and ${{\mathrm{Cpl}}}_{{{\mathrm{graph}}}}$ are the mean clustering coefficient and characteristic path length, respectively, of the graphs under examination, ${{\mathrm{Cc}}}_{{{\mathrm{rand}}}}$ and ${{\mathrm{Cpl}}}_{{{\mathrm{rand}}}}$ are the same measures on random graphs of the same size of the originating graphs. In particular, we have used uniform random graphs generated by the Erdos–Renyi (ER) model. The ER model that we have used has two parameters, the number of vertices $n$ and the number of edges $m$, where $0\le m\le {n}\left(n-2\right)/2$. For this study, the values of $n$ and $m$ that we have passed to the random-graph generator were the same as for the networks for which we wanted to determine the small-world-coefficient. In doing so—remarkably—the originating networks were compared to equivalent ER graphs with the same size and mean network degree, and the small-world-coefficient was determined on the basis of the differences ascribable to the sole network topology. The choice of using uniform ER random graphs within Eq. (5) for the derivation of the small world coefficient of the networks is not arbitrary, but is motivated by the seminal study reported in ref. ^17^, in which the Authors for the first time defined a precise measure of “small-world-ness” based on the trade-off between high local clustering and short path length. In their studies, they determined quantitatively (rather than through a categorical distinction) the small world status of a network *G* by comparing its topology metrics (i.e., the clustering coefficient and the characteristic path length) to the same measures performed on random ER graphs with the same size as *G*. Thus, the optimal value of small-world-ness of $4.8$ that we have determined in our study is valid under the null hypothesis of uniform Erdos–Renyi graphs used as a comparative sample. The use of different definitions for the random graphs in Eq. (5) would lead to different values of small-world-ness that optimize the network’s performance—similarly to the uncertainty that arises when estimating “network motifs”, as described in the beautiful comment reported in ref. ^29^.

Thus, Eqs. (4) and (5) provide a quantitative measure of the small-world-ness of a network based on the knowledge of Cc and Cpl^17^. Based on this definition, a graph $G$ has the attributes of small-world-ness if it has shortest paths and higher clustering than a random analog with the same size of $G$. The categorical definition of small-world network implies ${\rm{\varphi }}\ge 1$, ${\rm{\gamma }}\gg 1$, which, in turn, yields ${\rm{SW}}\, > \,1$.

### Simulating the propagation of a signal in the networks

To examine how a disturbance propagates in the systems, we modeled the networks as sets of artificial neurons. Each neuron is topologically and physically connected to a number of other neurons in consonance with the adjacency matrix of the network. When the initial stimulus, i.e., a sequence of current pulses (*I*_stim_), is applied to a node randomly picked from the network, it stimulates the neuron associated with that node causing over time a variation of its membrane potential ($V$) as:

6 $${C}_{{\mathrm{m}}}\frac{{{\mathrm{d}}V}}{{{\mathrm{d}}t}}=-{g}_{{\mathrm{l}}}\left(V-{V}_{{\mathrm{o}}}\right)+{I}_{{{\mathrm{stim}}}},$$

where *C*_m_, ${g}_{{\mathrm{l}}}={C}_{{\mathrm{m}}}/\tau$, and *V*_o_ are the capacitance, the conductance, and the resting potential of the membrane, and $\tau$ is the time constant of the system. In the simulations, we assigned the following values to the model parameters: ${C}_{{\mathrm{m}}}=300\,{{\mathrm{pF}}}$, $\tau =3\,{{\mathrm{ms}}}$, ${V}_{{\mathrm{o}}}=6\,{{\mathrm{mV}}}$. Moreover, *I*_stim_ was modeled as the repetitions of $N$ words, i.e., arrays of $8$ letters, where each letter is a binary variable of $0$ or $J$, with the probability of being $J$, $p$. Moreover, $J$ is the signal strength and is such that $J/{C}_{{\mathrm{m}}}=0.25\,{{\mathrm{mV}}}$. In this study, $N$ was varied between $1$ and $100$ ($N=1,2,3,4,5,10,20,50,100$), and $p$ between $0.1$ and $1$. $N$ and $p$ are proportional to the length and the frequency of the signal. Equation (6) is a version of the generalized leaky integrate and fire model^30,31^, and describes how the potential at the postsynaptic sites of a neuron varies with time following an intermittent stimulus.

Each time that the neuron response exceeds a value of threshold (*V*_th_ = 9 mV) the neuron produces an action potential (AP). The sequence of action potentials that the neuronal unit ($i$) produces over time represents the output signal of that unit and takes the form: $J\mathop{\sum }\nolimits_{k}^{{APN}}\delta \left(t-{t}_{i}^{k}\right)$. Where: $J$ is the amplitude of the output current, $\delta$ is Kronecker delta, being $1$ when the time of the process ($t$) matches the characteristic pattern of AP events of the neuron (${t}_{i}^{k}$), and $0$ otherwise. APN is the total number of action potentials produced by the neuron. Thus, the response of a neuronal unit is quantized in time.

Individual neuronal responses then converge into the unit ($j$) to which they are connected, producing in turn a current of stimulus: ${I}_{{{\mathrm{stim}}}}\left(t\right)=\mathop{\sum }\nolimits_{i}^{{\mathscr{B}}}\zeta \left({d}_{{ij}}\right){J}\mathop{\sum }\nolimits_{k}^{{{\mathrm{rel}}}}\delta \left(t-{t}_{i}^{k}\right)$. In this equation, *I*_stim_ is the sum of currents $J$ over all the neurons $i$ insisting on neuron $j$ (${\mathscr{B}}$), and $\zeta$ is a damping factor that accounts for the attenuation of a signal traveling from $i$ to $j$ through the distance ${d}_{{ij}}$. The response and behavior of the target neuron $j$ (at any time at the leading edge of the perturbation) is determined again using Eq. (6). Thus, the output of a unit is passed repeatedly as an input to the other units with which it has established some connections. The repetition of this mechanism generates a wave of signals that travels in the grid depending on the grid characteristics. Equation (6) was solved numerically using forward Euler integrations of the finite-difference equations resulting from the discretization of the derivative operators. The time integration uses an explicit trapezoidal scheme and we assume null initial conditions. The initial mesh consists of $400$ grid points. The time step used is $\Delta t=0.01\,{{\mathrm{ms}}}$.

### Measuring the information transported in the networks

The patterns of action potentials produced at each node of the network are represented in our model as sequences of $0$ and $1$ (bits). We used the methods of information theory to decode the information contained in these arrays^20,21,23^. To do so, we partitioned lists of values in subsets called words. A word is a finite sequence of $8$ bits. Thus, each word can represent a signal in ${2}^{8}=256$ independent combinations. Then, we found for each word the number of times it is represented in the originating list, from which we derived the frequency distributions of words, or substates, in the system’s response, sorted in order of decreasing frequencies: $P(s)$. At this point, we found the Shannon information entropy $H$ be associated to $P(s)$^20^ as $H\left(S\right)=-\sum _{S}P(s){{\mathrm{log}}}_{2}P(s)$, where $s$ stands for state and $S$ for stimulus. $H$ quantifies the average amount of information gained with each stimulus presentation. To calculate the net information transported in the networks, we applied $H$ to a random input signal ($T$) and a periodic stimulus ($N$), and calculated the difference: $I=T-N$. Exhaustive details on the methods are reported in ref. ^18^.

## Supplementary information

Supplementary Material

## Data Availability

All data and related metadata underlying reported findings and the algorithm utilized in the study are deposited in the public data repository OSF under the name “Small World Information” (10.17605/OSF.IO/AS65R).
