# Supplementary material for: The small world coefficient 4.8 ± 1 optimizes information processing in 2D neuronal networks
Source: NPJ Syst Biol Appl. 2022 Jan 27;8:4. doi: 10.1038/s41540-022-00215-y (PMC8795235; doi:10.1038/s41540-022-00215-y)
Supplement: Supplementary file 1 — Supplementary Material [file 41540_2022_215_MOESM1_ESM.pdf]

The Small World coefficient 4.8±1 optimizes information processing in 2D neuronal networks

F. Aprile [1], V. Onesto [2], F. Gentile [3,*]

[1] Department of Electric Engineering and Information Technology, University Federico II, 80125 Naples, Italy

[2] Institute of Nanotechnology, National Research Council (CNR-NANOTEC), Campus Ecotekne, via Monteroni, Lecce, 73100 Italy

[3] Nanotechnology Research Center, Department of Experimental and Clinical Medicine, University of Magna Graecia, 88100 Catanzaro, Italy

* Author to whom correspondence should be addressed: francesco.gentile@unicz.it

Supplementary Information

Supplementary Information section 1.
Examples of networks obtained by wiring points in the plane by distance rule and find of density peaks algorithm. Total number and distribution of the configurations generated with this method. — *Pg. 2*

Supplementary Information section 2.
All data relative to the active nodes (η^{nodes}), total information (η^{grid}) and peak information (η^{peak}) enhancement factors as a function of the small-world-ness of the networks of neuronal cells, for different values of length and frequency of the input signal. — *Pg. 3*

Supplementary Information section 3.
Values of quality factor associated to η^{grid}, η^{peak} and η^{nodes} calculated for different combinations of signal length (Δt) and signal frequency (f), and for values of the small world coefficient spanning the $1-14$ interval. — *Pg. 23*

Supplementary Information section 4.
Network analysis of graphs. — *Pg. 32*

Supplementary Information section 1. Examples of networks obtained by wiring points in the plane by distance rule and find of density peaks algorithm. Total number and distribution of the configurations generated with this method.

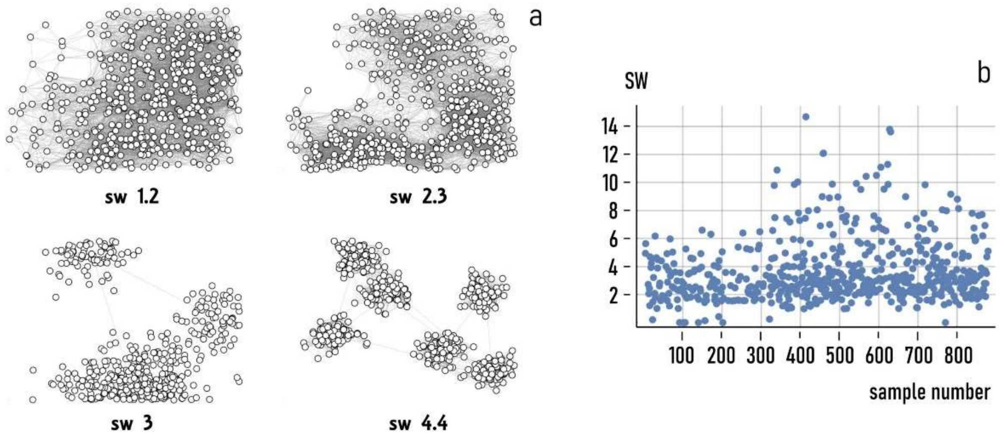

Supplementary Figure 1. Using an algorithm that connects points on the basis of their distance and local density, we generated networks with different values of small-world-ness, in the example: $\mathrm{SW} = 1.2$, $\mathrm{SW} = 2.3$, $\mathrm{SW} = 3$, $\mathrm{SW} = 4.4$ (***a***). Applying repeatedly the algorithms to points sampled from Gaussian distributions, we obtained ~ 1000 configurations with small world coefficients comprised in the $0 - 14$ range (***b***).

Supplementary Information section 2. All data relative to η^{nodes}, η^{grid} and η^{peak} as a function of the small-world-ness of the networks of neuronal cells, for different values of length and frequency of the input signal.

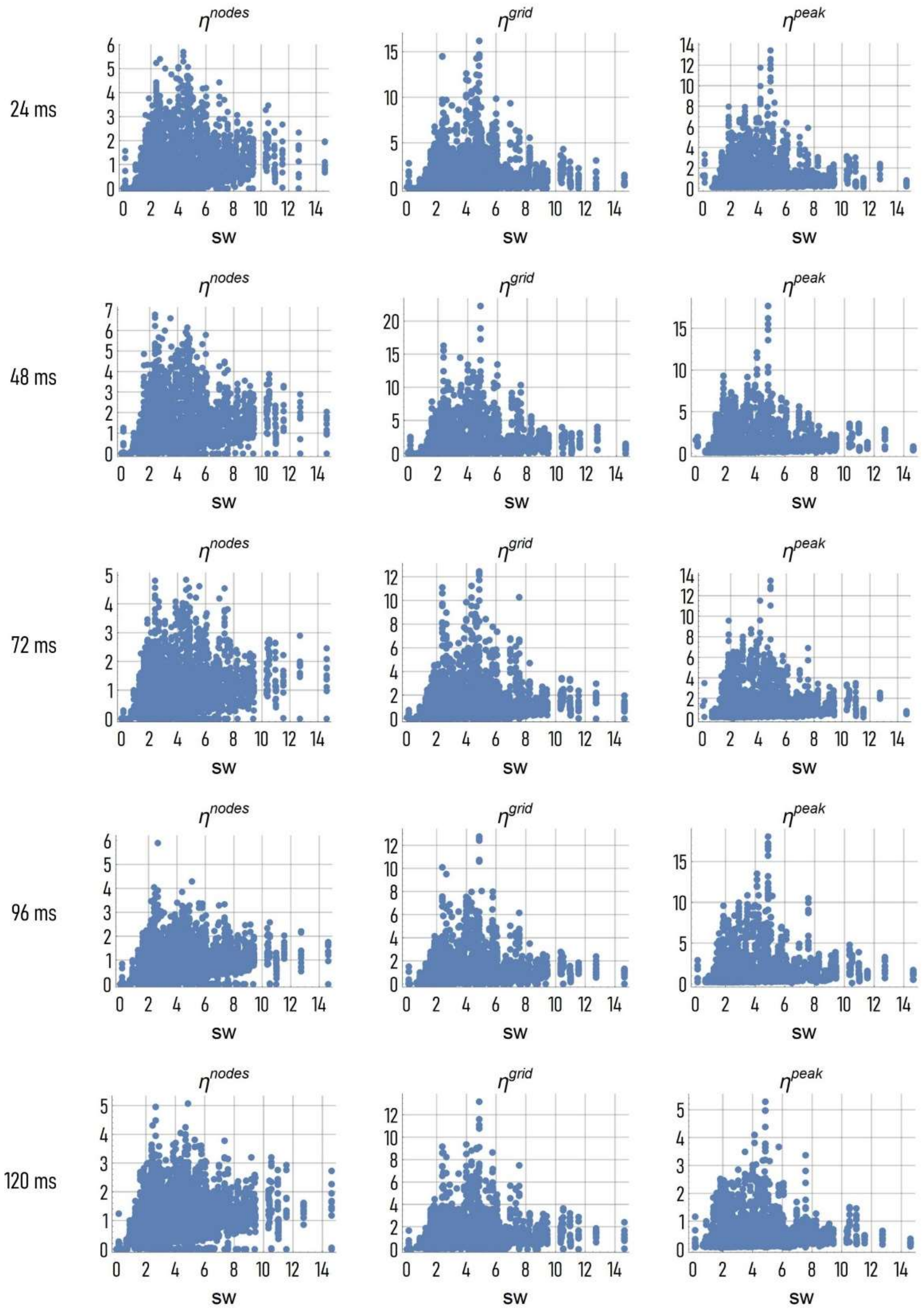

Supplementary Figure 2. Measured values of η^{nodes}, η^{grid} and η^{peak} as a function of the small world coefficient SW, measured for a signal length $\Delta t = 24,\ 48, 72, 96, 120\ ms$, and a frequency $f = 33\ Hz$.

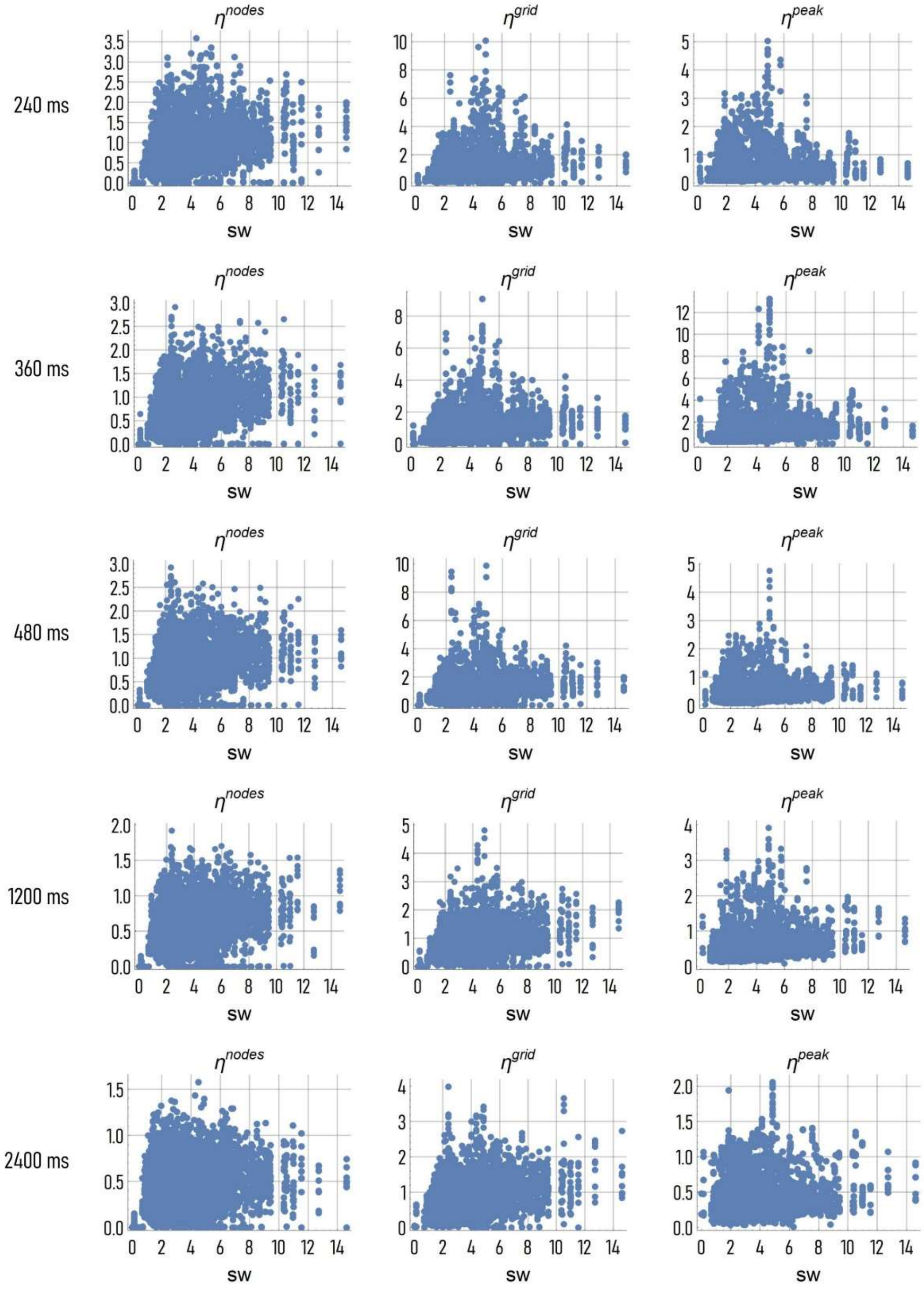

Supplementary Figure 3. Measured values of η^{nodes}, η^{grid} and η^{peak} as a function of the small world coefficient SW, measured for a signal length $\Delta t = 240,\ 360, 480, 1200, 2400\ ms$, and a frequency $f = 33\ Hz$.

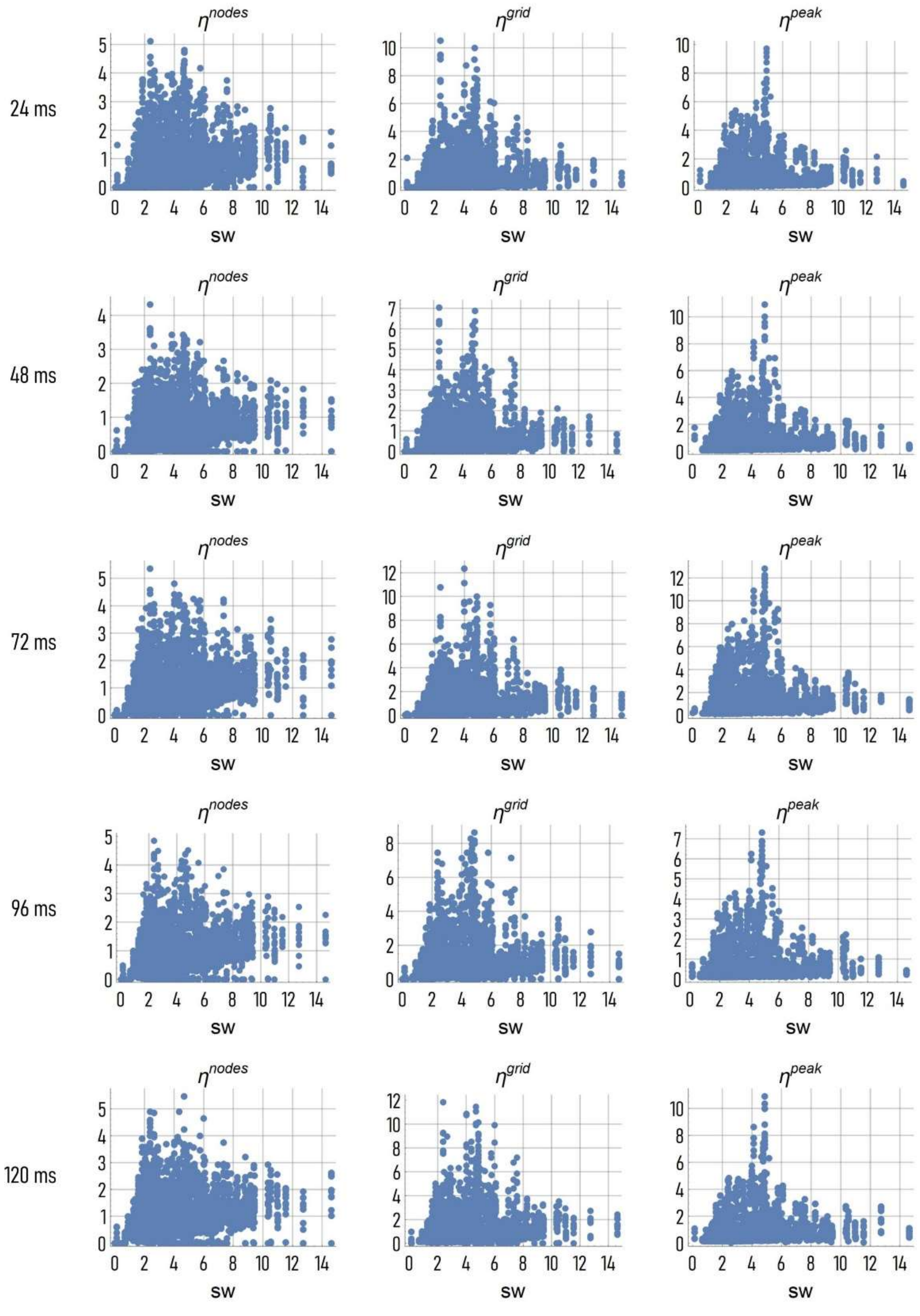

Supplementary Figure 4. Measured values of η^{nodes}, η^{grid} and η^{peak} as a function of the small world coefficient SW, measured for a signal length $\Delta t = 24,\ 48, 72, 96, 120\ ms$, and a frequency $f = 67\ Hz$.

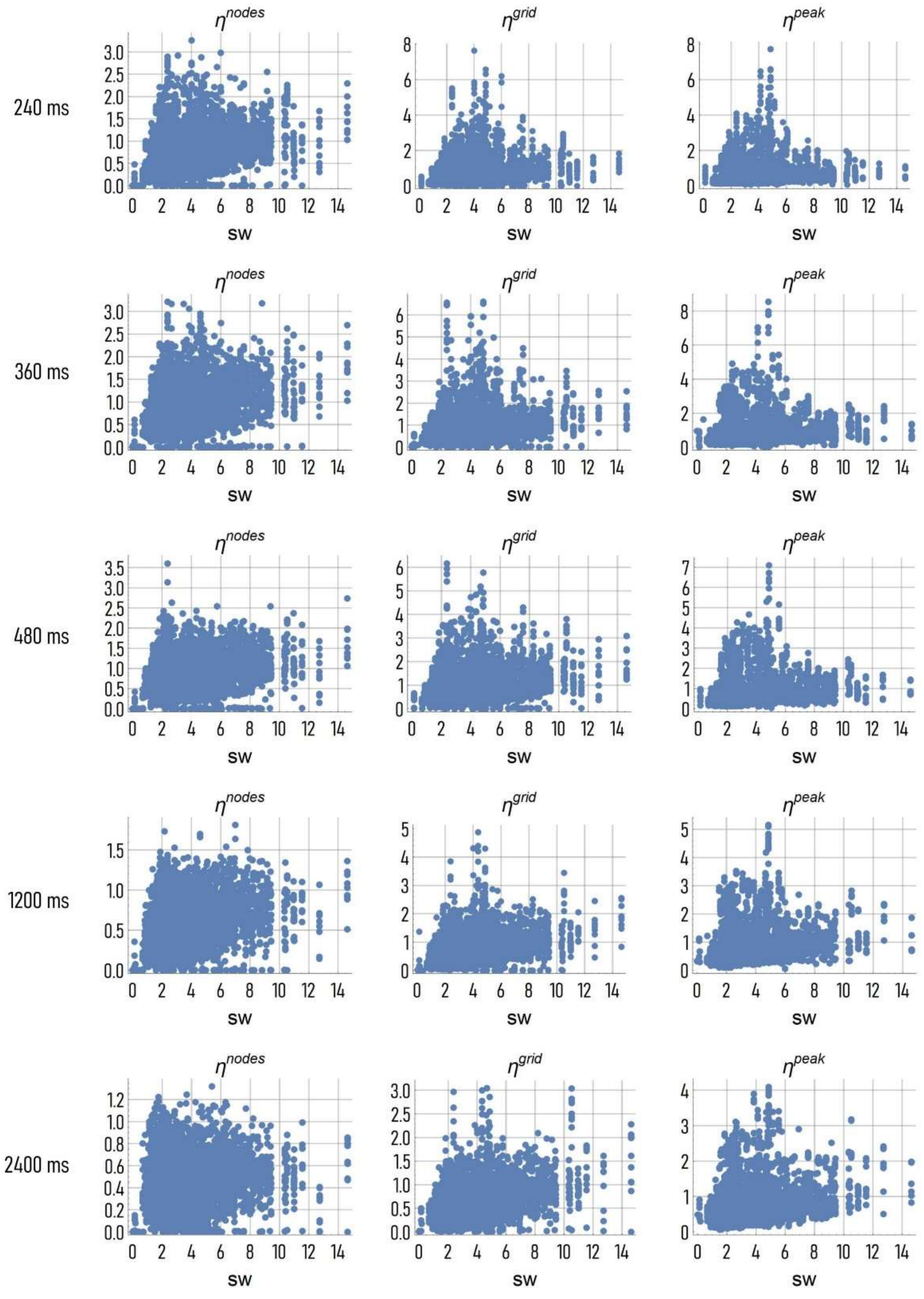

Supplementary Figure 5. Measured values of η^{nodes}, η^{grid} and η^{peak} as a function of the small world coefficient SW, measured for a signal length $\Delta t = 240,\ 360, 480, 1200, 2400\ ms$, and a frequency $f = 67\ Hz$.

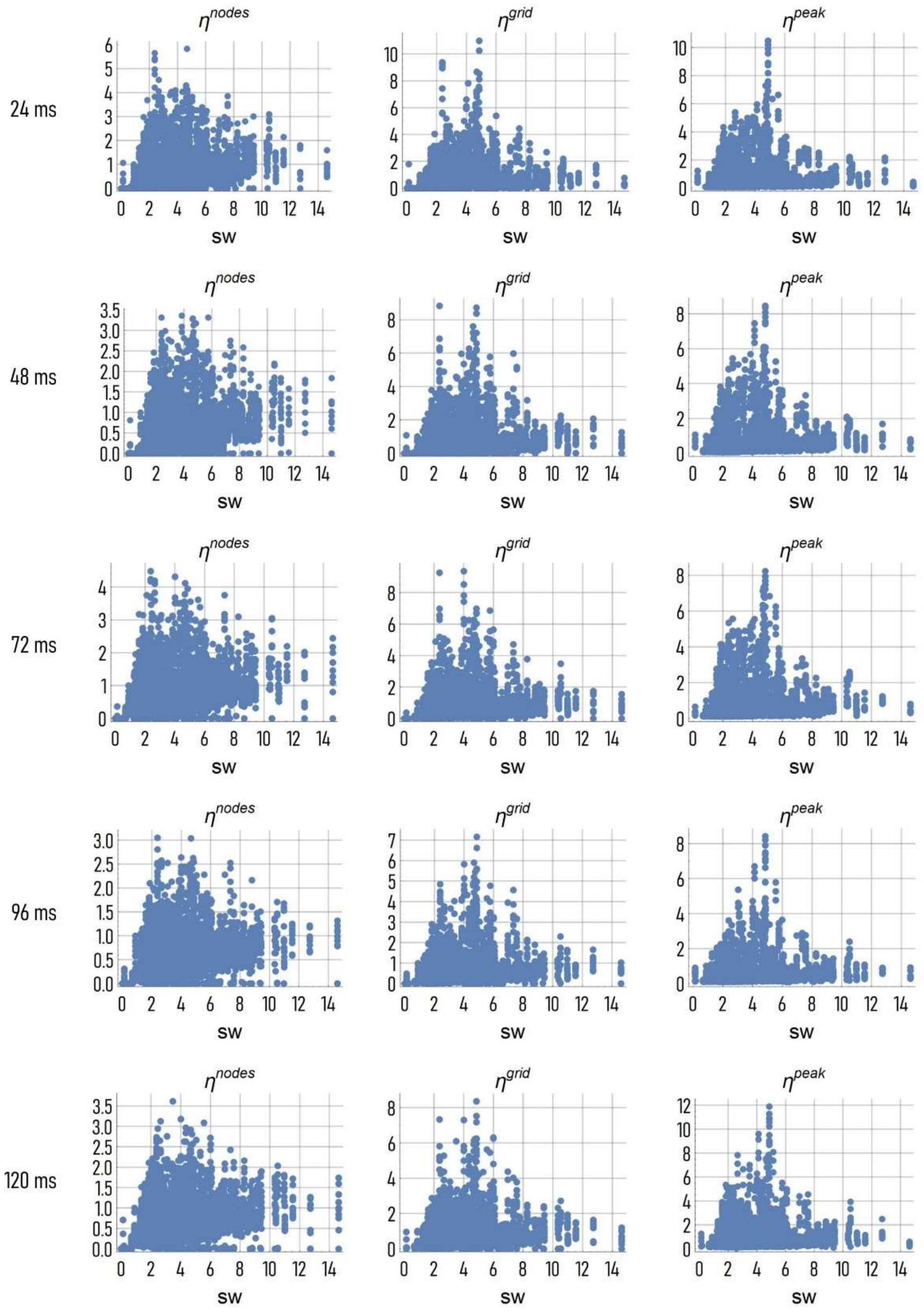

Supplementary Figure 6. Measured values of η^{nodes}, η^{grid} and η^{peak} as a function of the small world coefficient SW, measured for a signal length $\Delta t = 24,\ 48, 72, 96, 120\ ms$, and a frequency $f = 100\ Hz$.

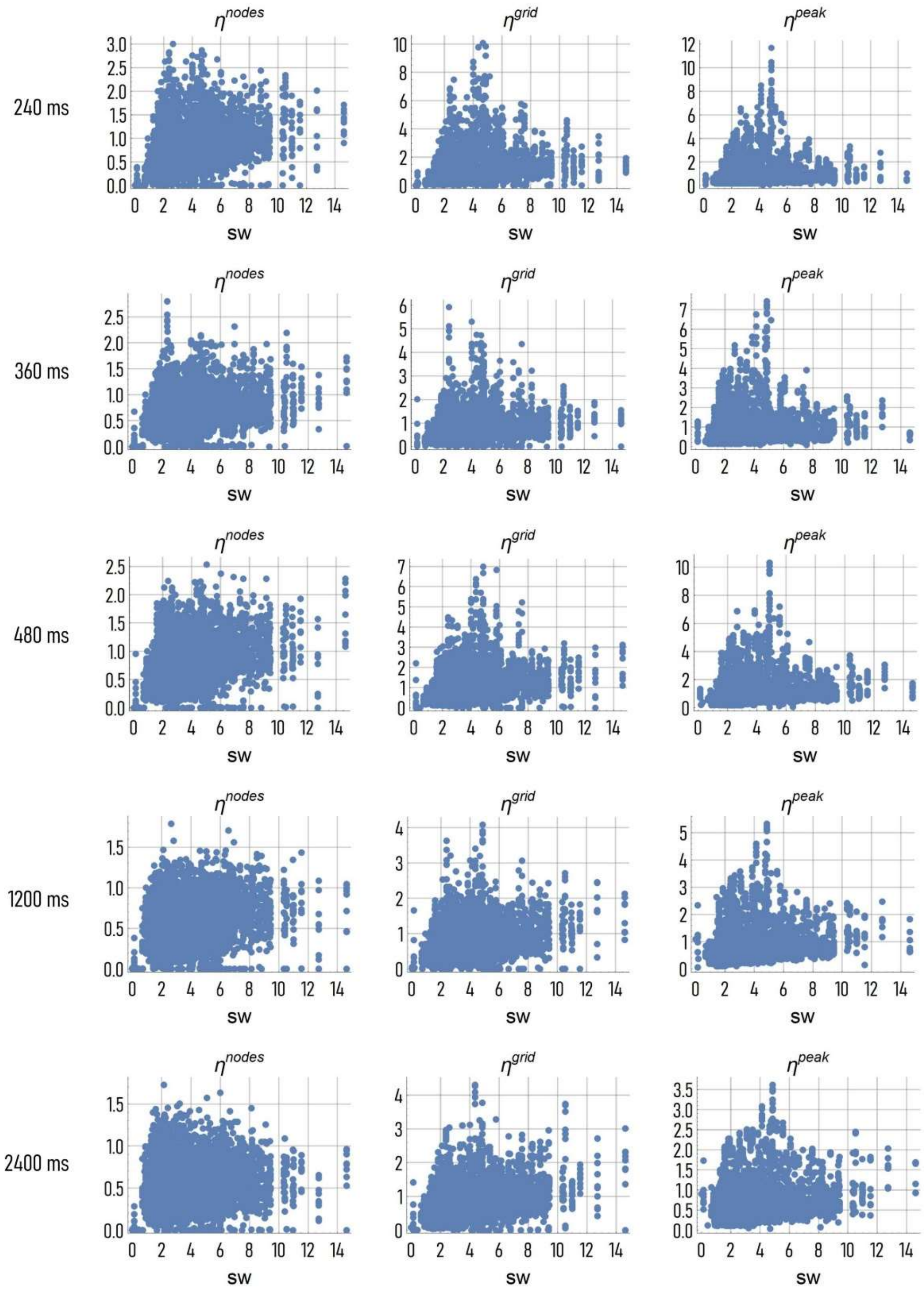

Supplementary Figure 7. Measured values of η^{nodes}, η^{grid} and η^{peak} as a function of the small world coefficient SW, measured for a signal length $\Delta t = 240,\ 360, 480, 1200, 2400\ ms$, and a frequency $f = 100\ Hz$.

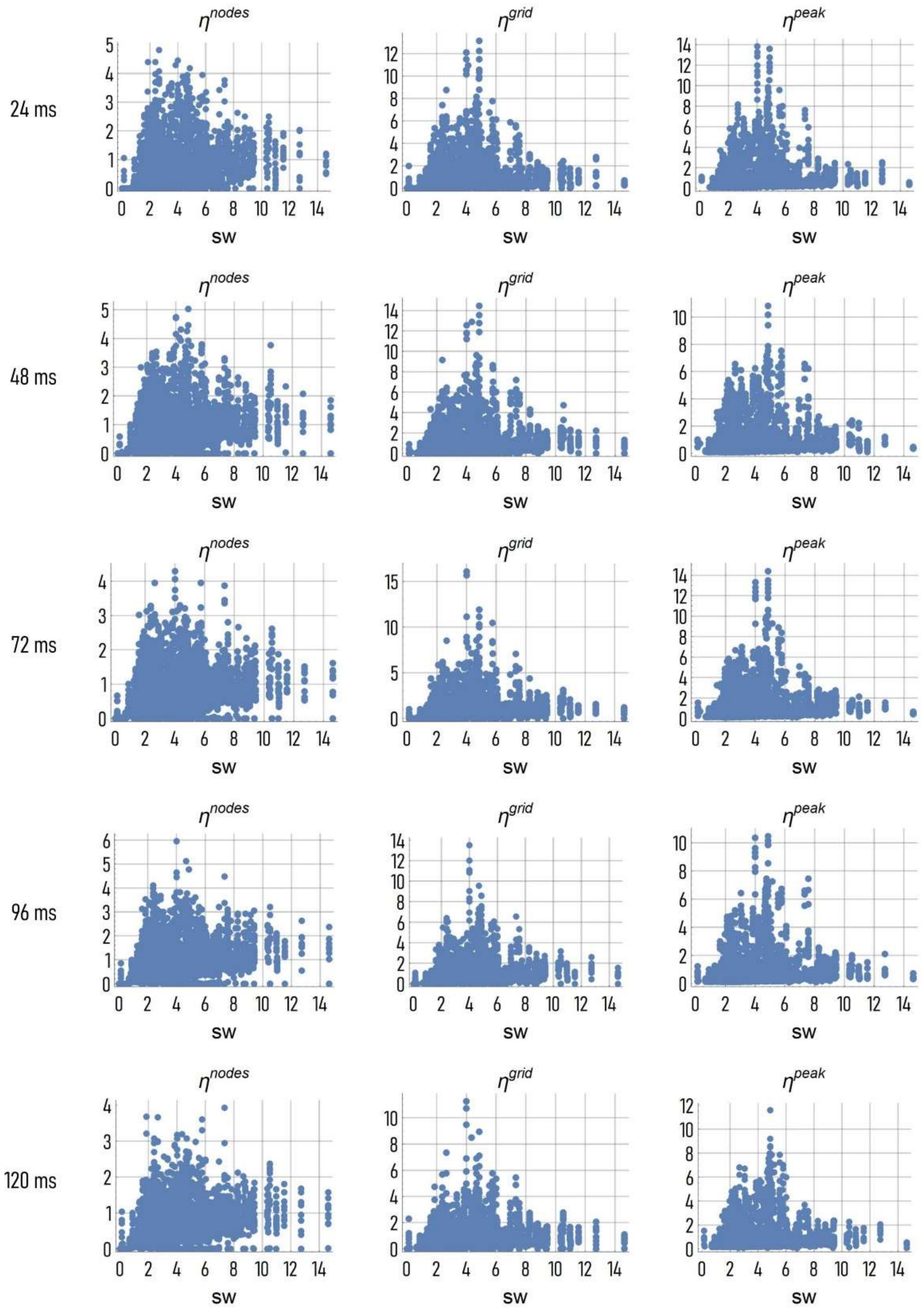

Supplementary Figure 8. Measured values of η^{nodes}, η^{grid} and η^{peak} as a function of the small world coefficient SW, measured for a signal length $\Delta t = 24,\ 48, 72, 96, 120\ ms$, and a frequency $f = 133\ Hz$.

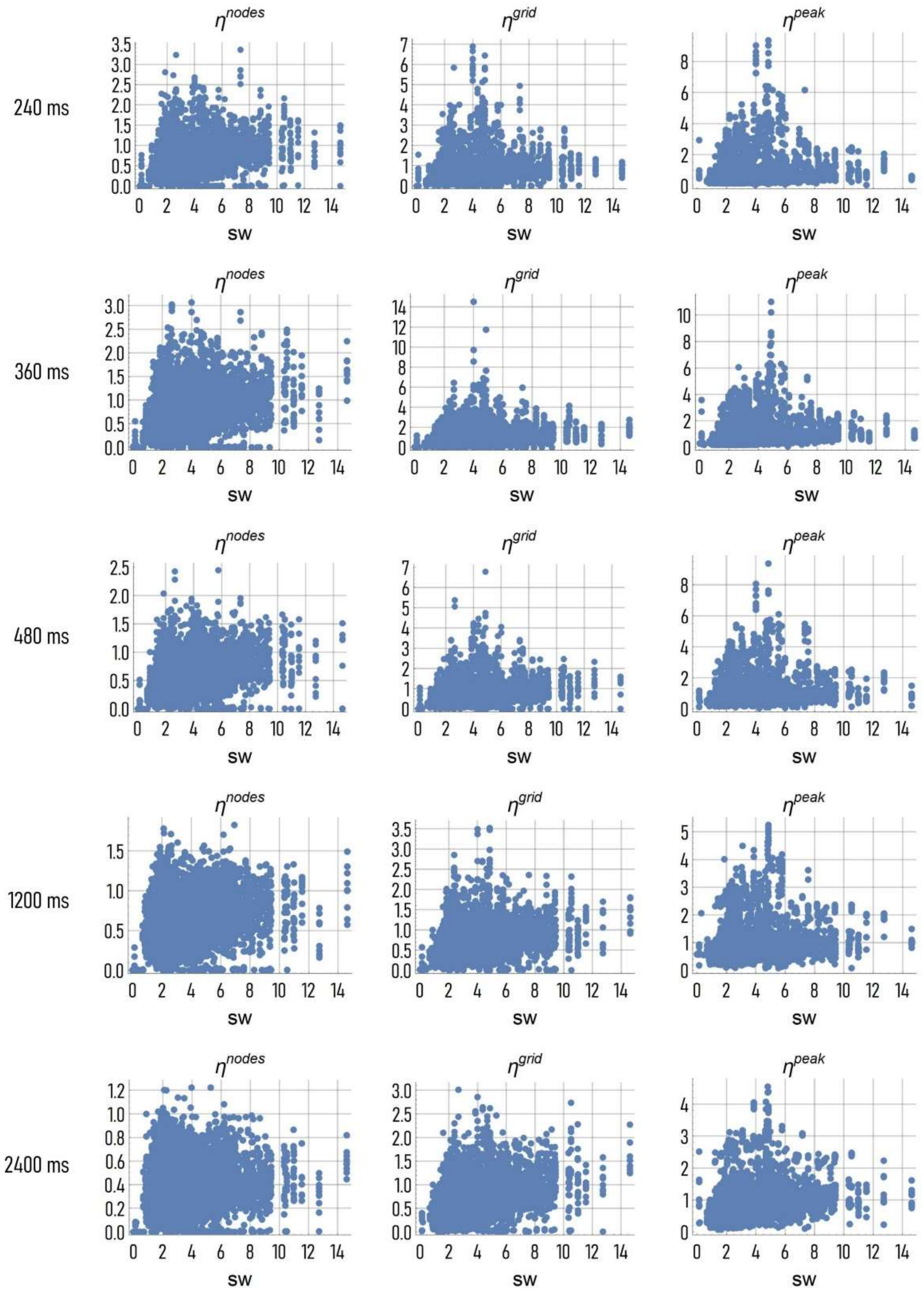

Supplementary Figure 9. Measured values of η^{nodes}, η^{grid} and η^{peak} as a function of the small world coefficient SW, measured for a signal length $\Delta t = 240,\ 360, 480, 1200, 2400\ ms$, and a frequency $f = 133\ Hz$.

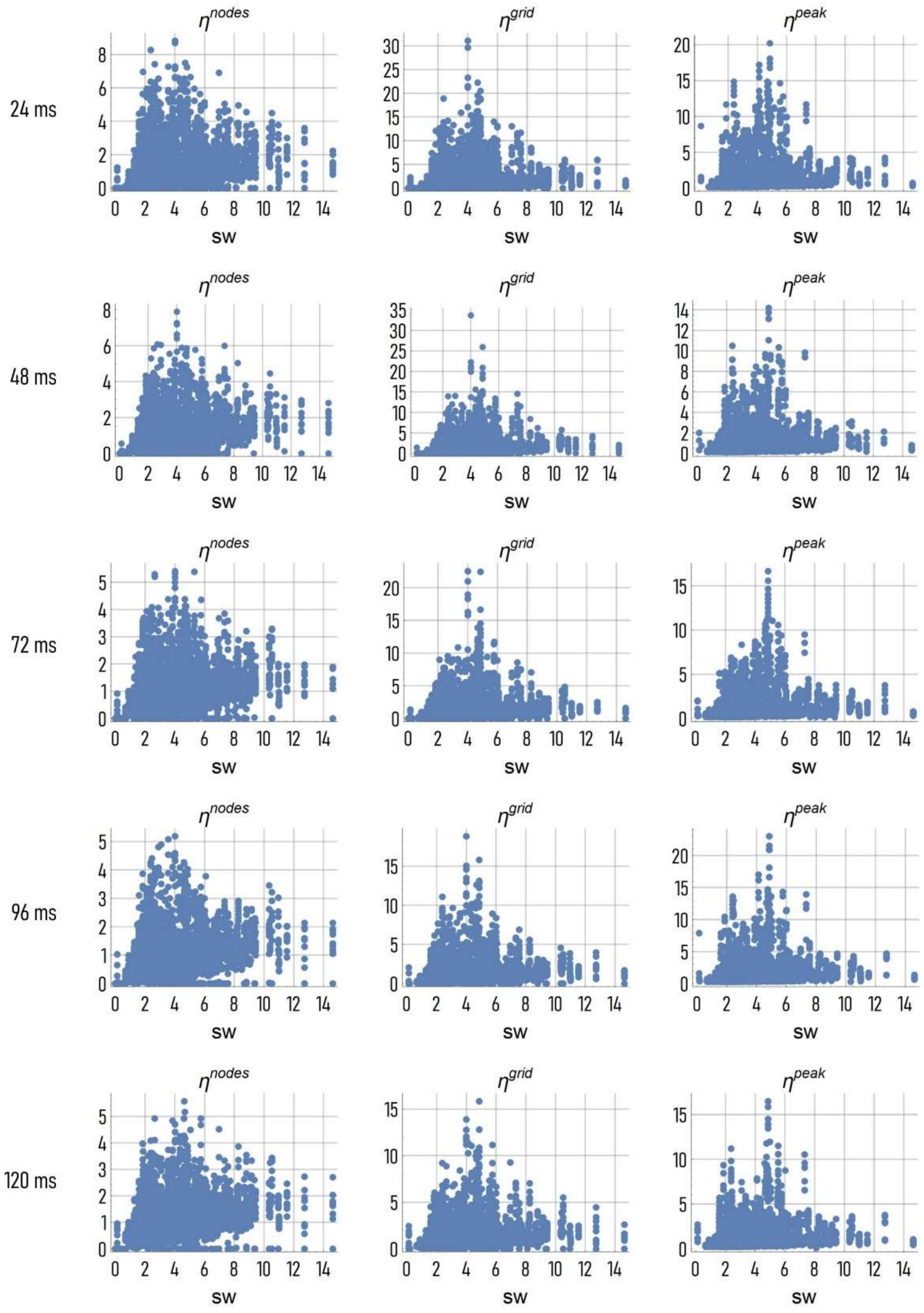

Supplementary Figure 10. Measured values of η^{nodes}, η^{grid} and η^{peak} as a function of the small world coefficient SW, measured for a signal length $\Delta t = 24,\ 48, 72, 96, 120\ ms$, and a frequency $f = 167\ Hz$.

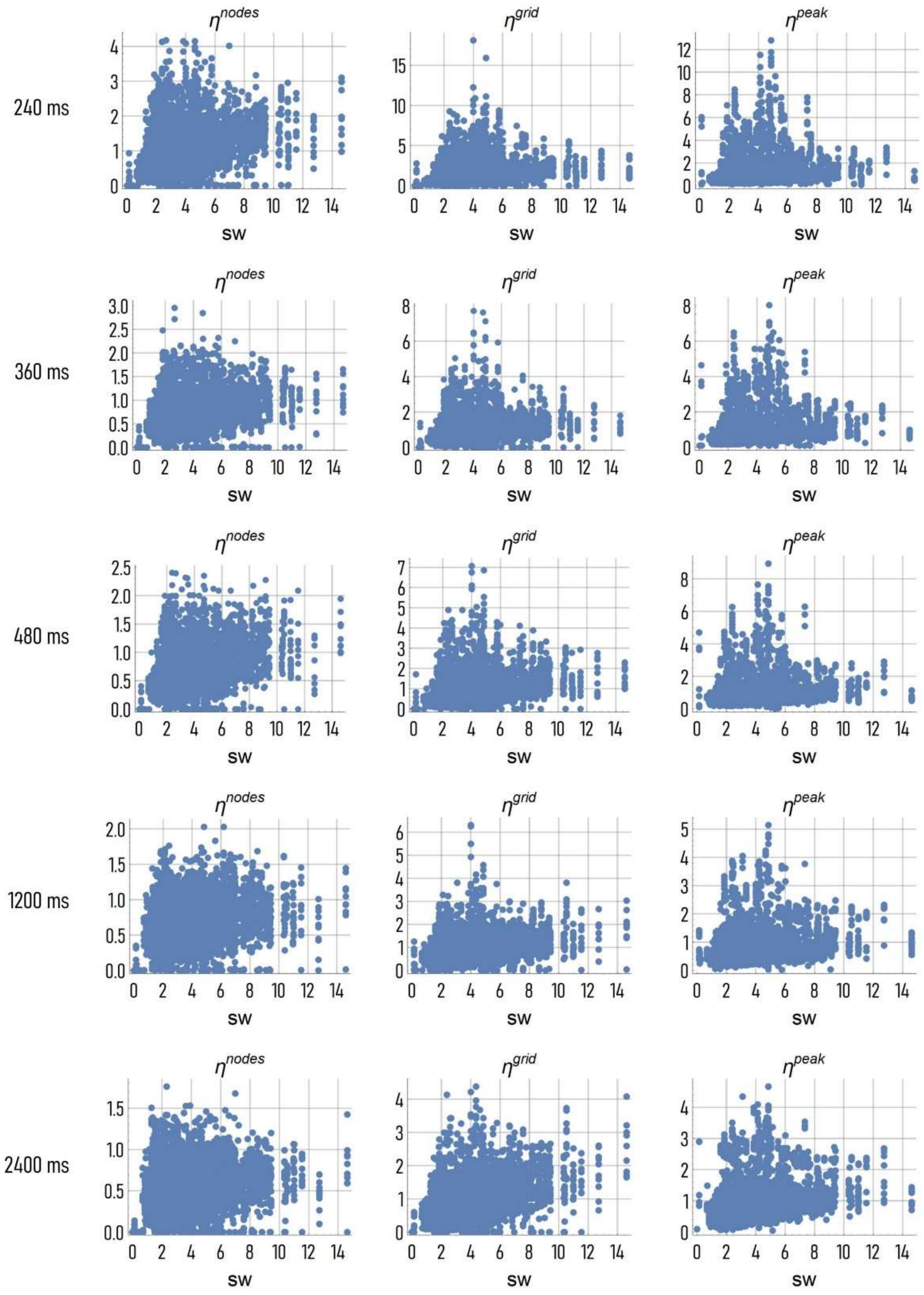

Supplementary Figure 11. Measured values of η^{nodes}, η^{grid} and η^{peak} as a function of the small world coefficient SW, measured for a signal length $\Delta t = 240,\ 360, 480, 1200, 2400\ ms$, and a frequency $f = 167\ Hz$.

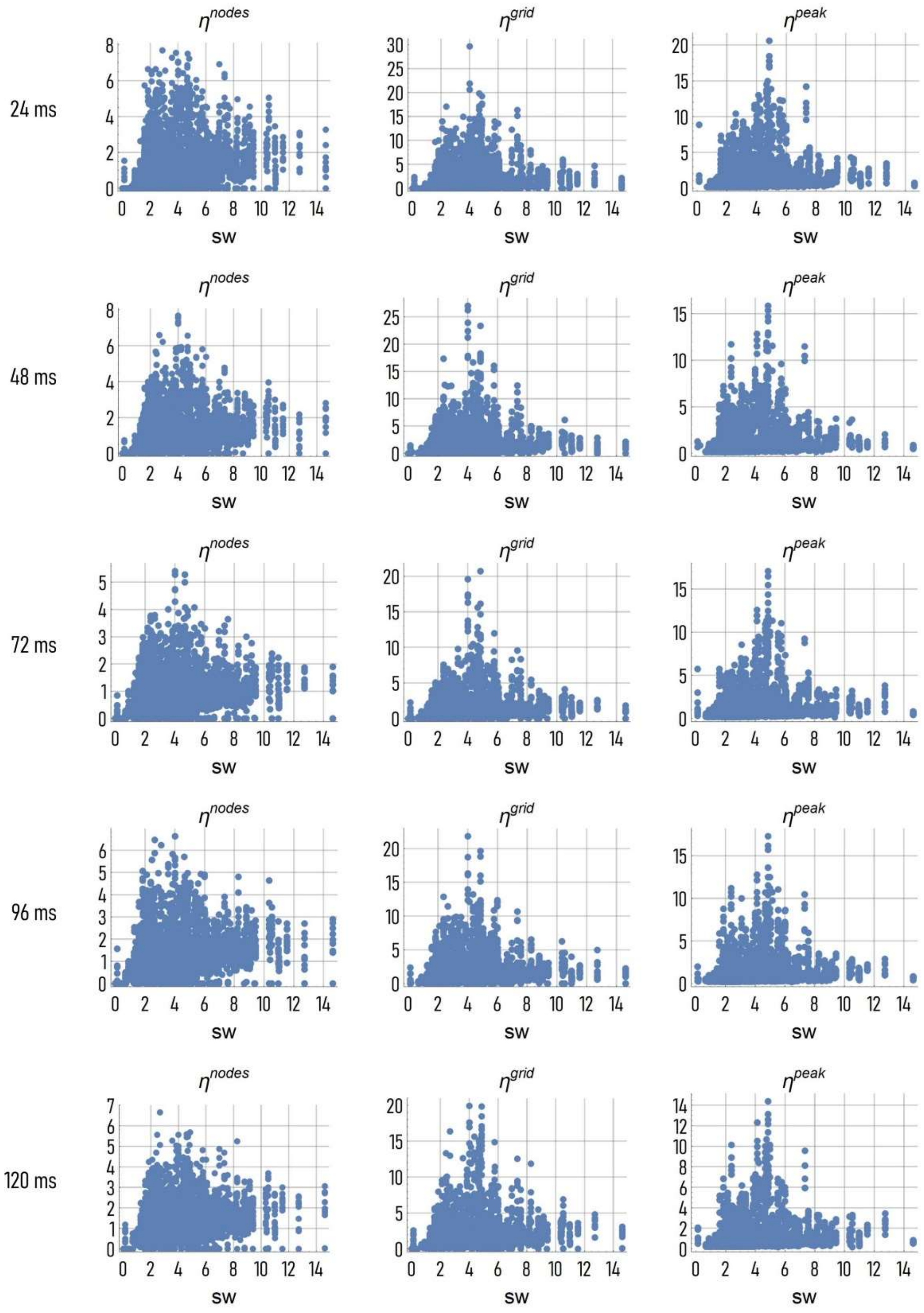

Supplementary Figure 12. Measured values of η^{nodes}, η^{grid} and η^{peak} as a function of the small world coefficient SW, measured for a signal length $\Delta t = 24,\ 48, 72, 96, 120\ ms$, and a frequency $f = 200\ Hz$.

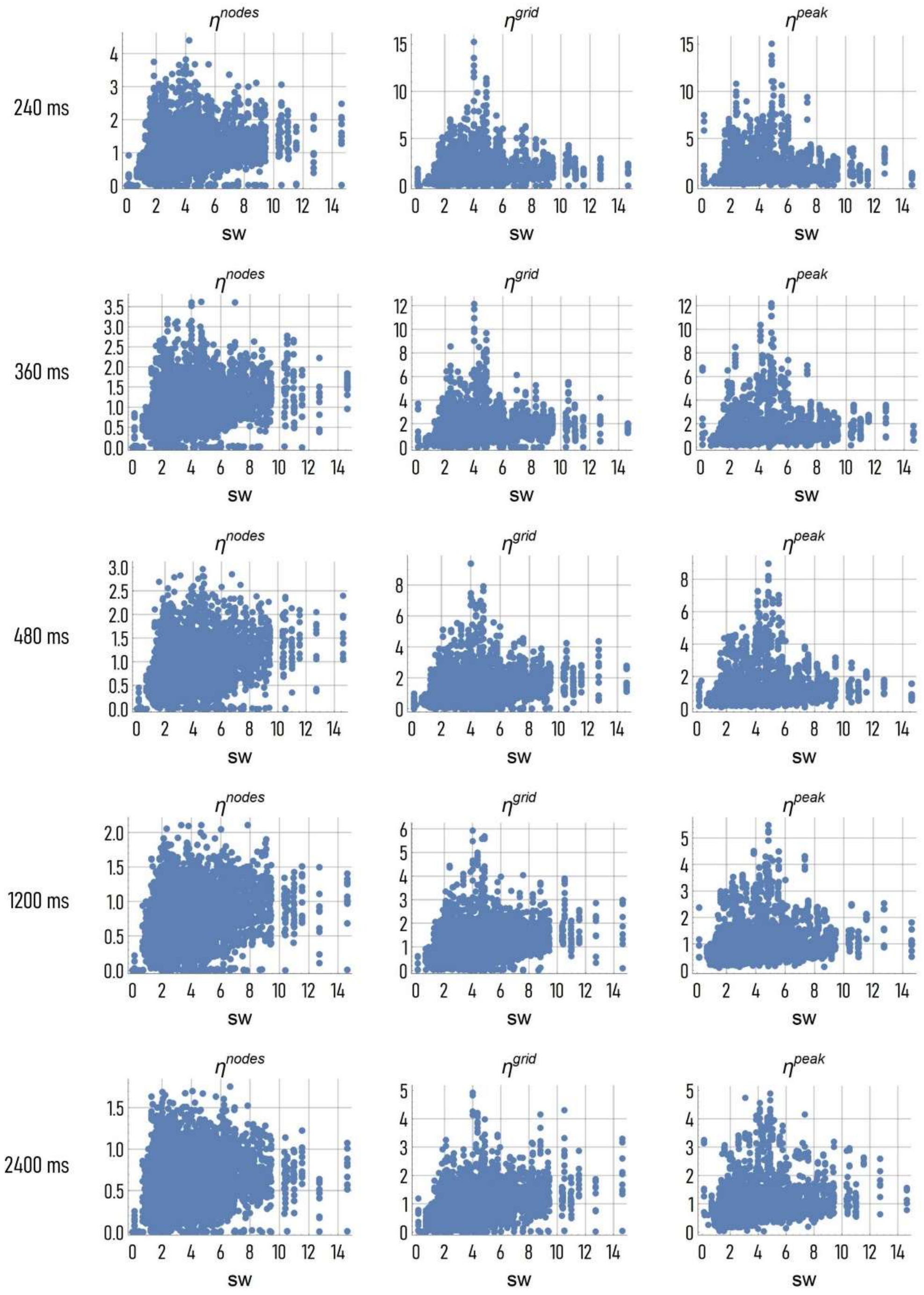

Supplementary Figure 13. Measured values of η^{nodes}, η^{grid} and η^{peak} as a function of the small world coefficient SW, measured for a signal length $\Delta t = 240,\ 360, 480, 1200, 2400\ ms$, and a frequency $f = 200\ Hz$.

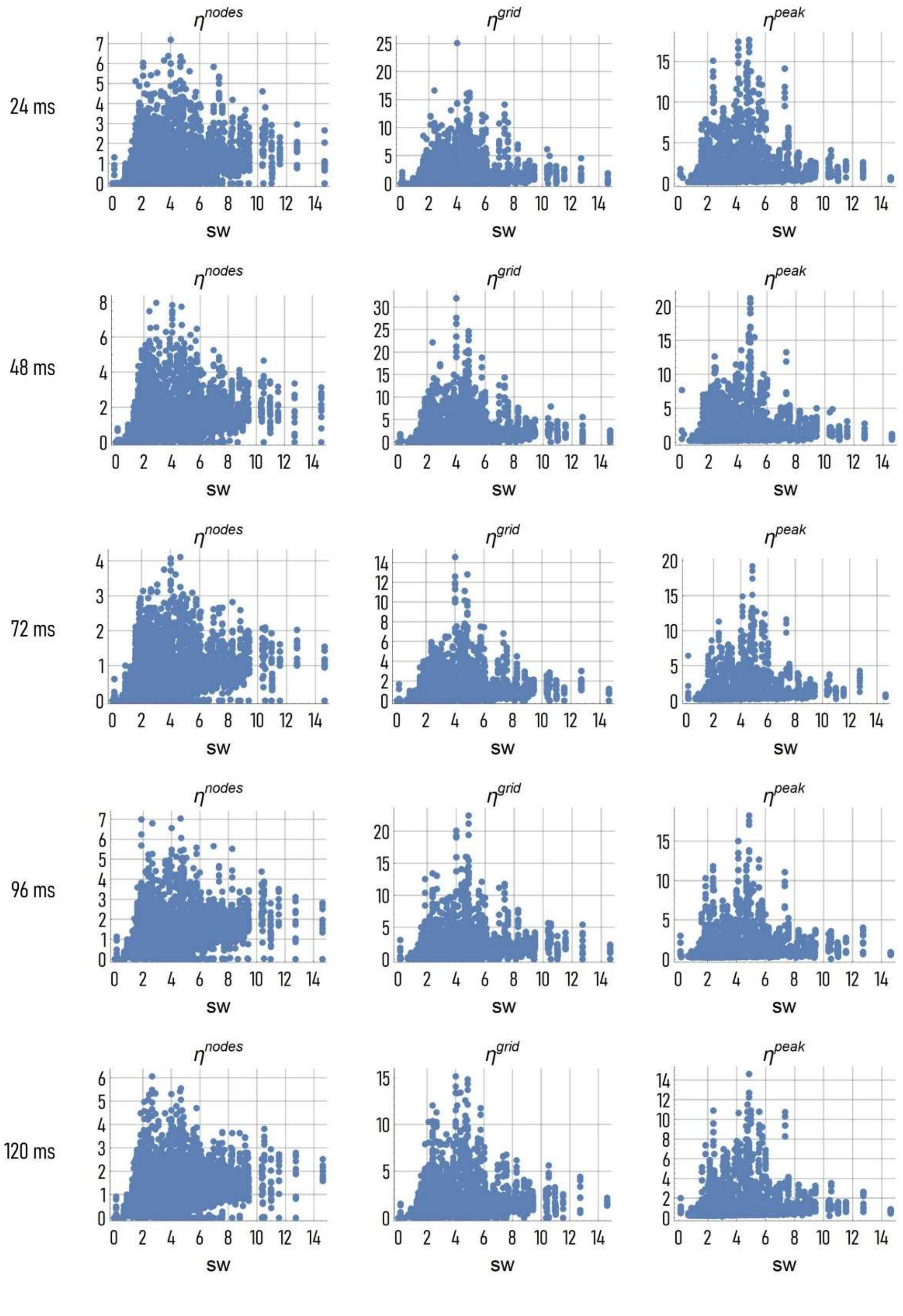

Supplementary Figure 14. Measured values of η^{nodes}, η^{grid} and η^{peak} as a function of the small world coefficient SW, measured for a signal length $\Delta t = 24,\ 48, 72, 96, 120\ ms$, and a frequency $f = 233\ Hz$.

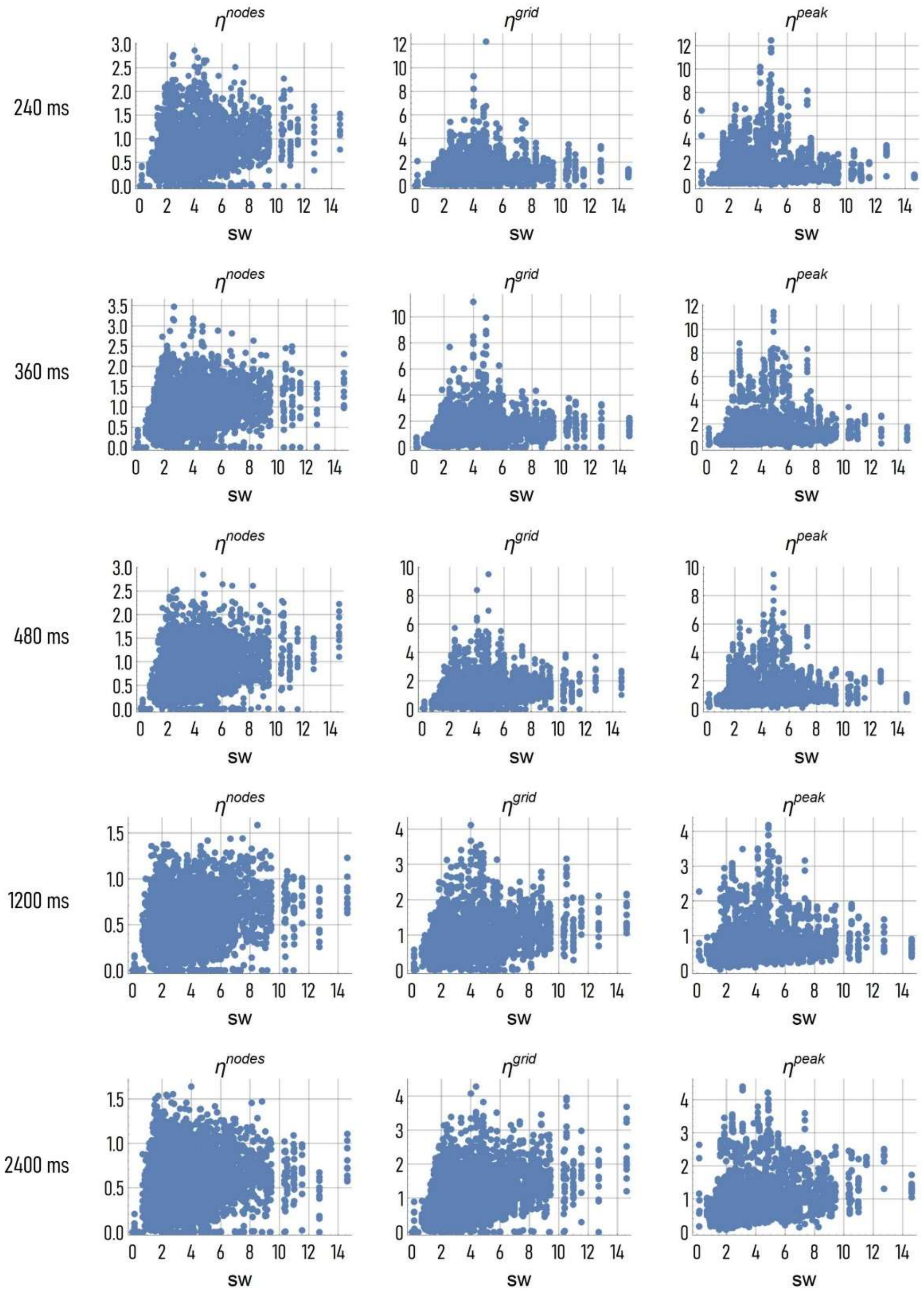

Supplementary Figure 15. Measured values of η^{nodes}, η^{grid} and η^{peak} as a function of the small world coefficient SW, measured for a signal length $\Delta t = 240,\ 360, 480, 1200, 2400\ ms$, and a frequency $f = 233\ Hz$.

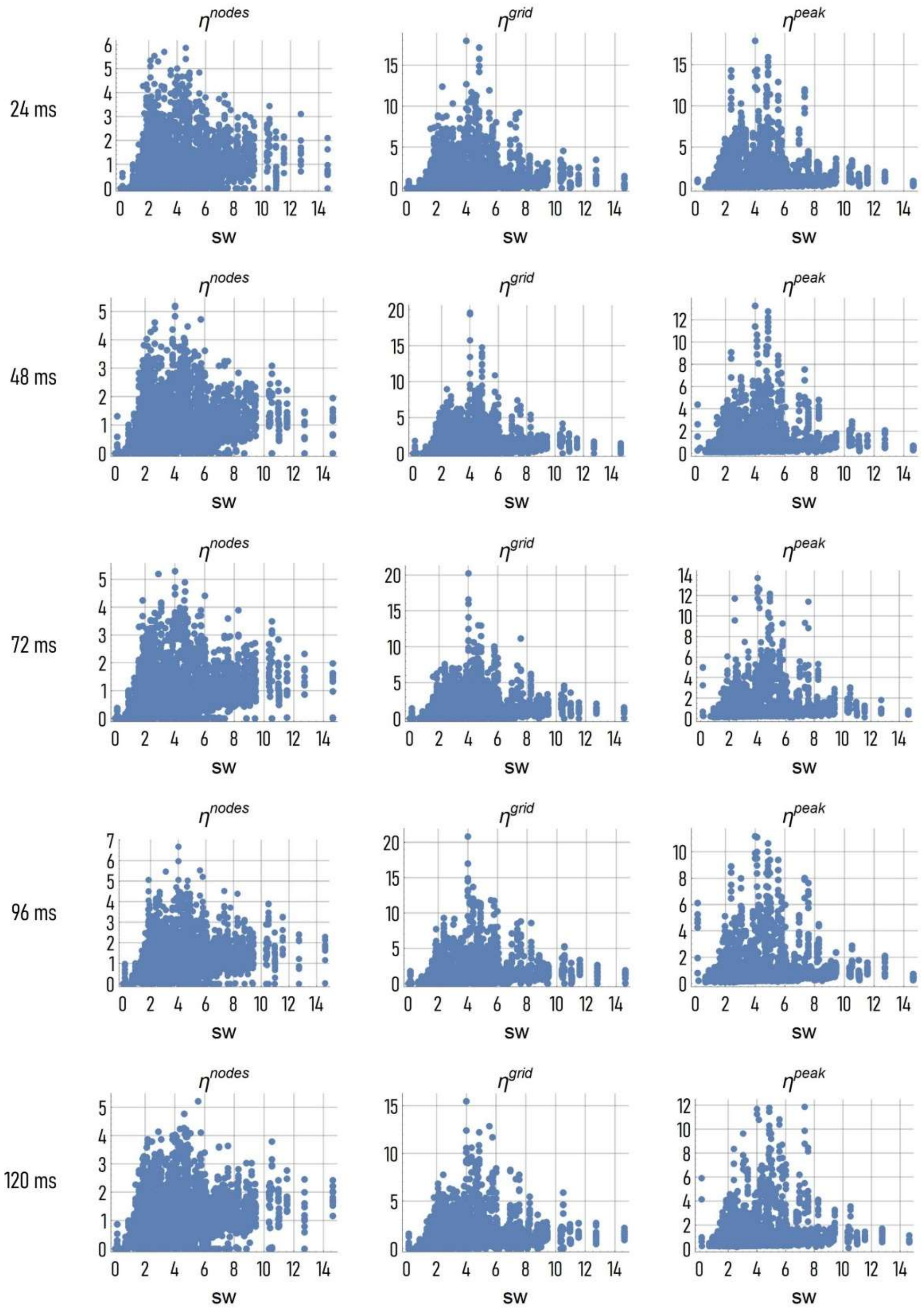

Supplementary Figure 16. Measured values of η^{nodes}, η^{grid} and η^{peak} as a function of the small world coefficient SW, measured for a signal length $\Delta t = 24,\ 48, 72, 96, 120\ ms$, and a frequency $f = 267\ Hz$.

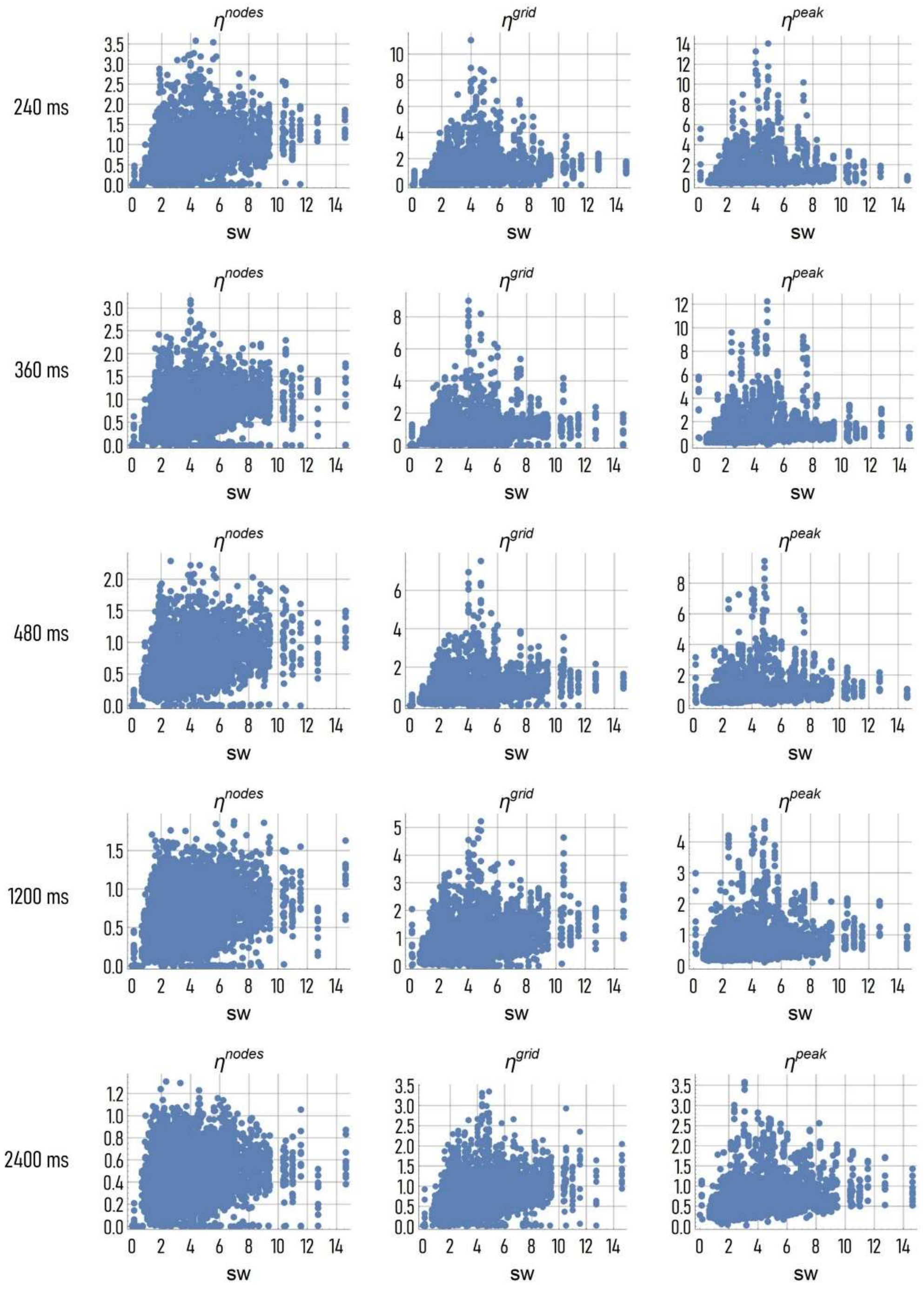

Supplementary Figure 17. Measured values of η^{nodes}, η^{grid} and η^{peak} as a function of the small world coefficient SW, measured for a signal length $\Delta t = 240,\ 360, 480, 1200, 2400\ ms$, and a frequency $f = 267\ Hz$.

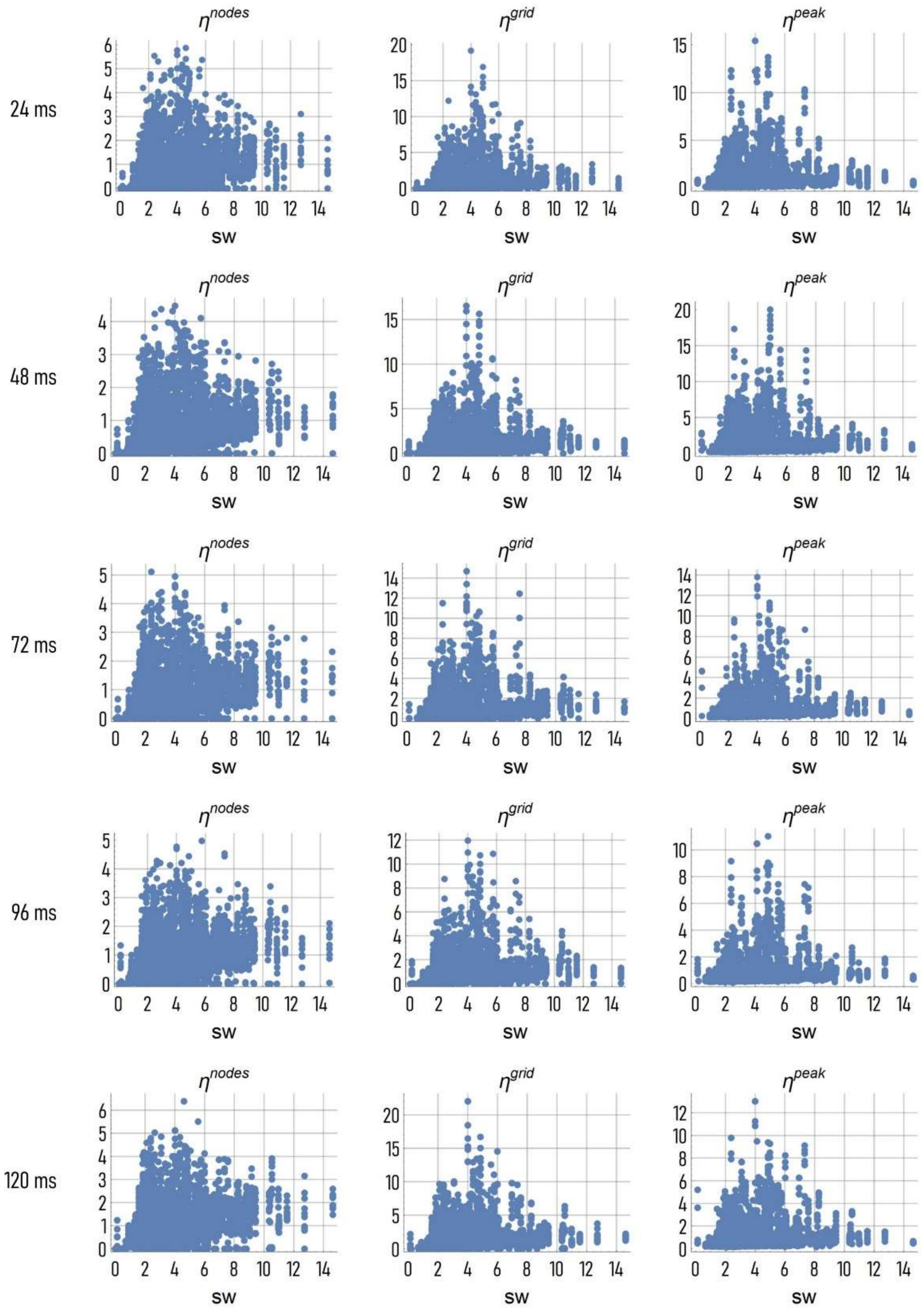

Supplementary Figure 18. Measured values of η^{nodes}, η^{grid} and η^{peak} as a function of the small world coefficient SW, measured for a signal length $\Delta t = 24,\ 48, 72, 96, 120\ ms$, and a frequency $f = 300\ Hz$.

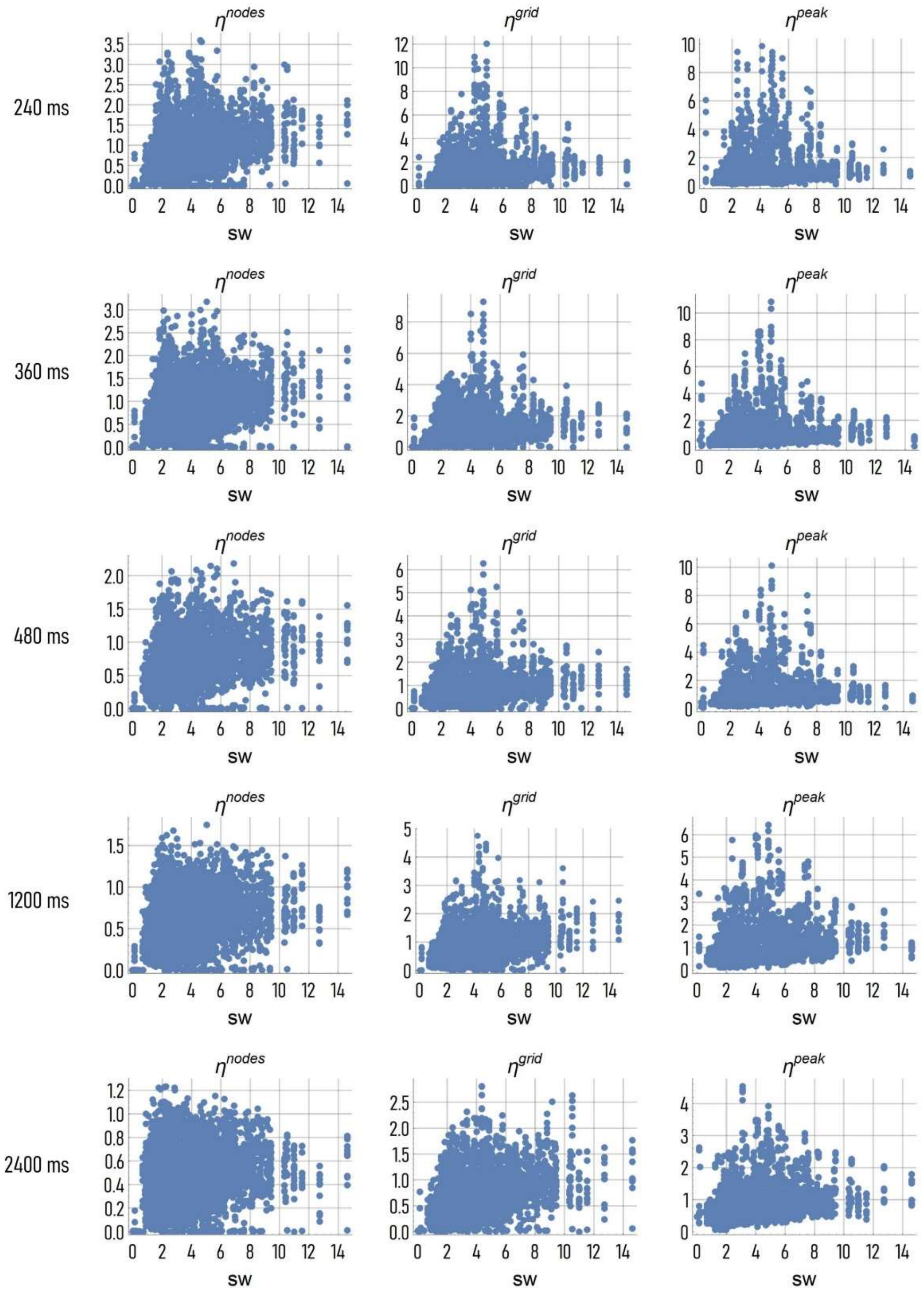

Supplementary Figure 19. Measured values of η^{nodes}, η^{grid} and η^{peak} as a function of the small world coefficient SW, measured for a signal length $\Delta t = 240,\ 360, 480, 1200, 2400\ ms$, and a frequency $f = 300\ Hz$.

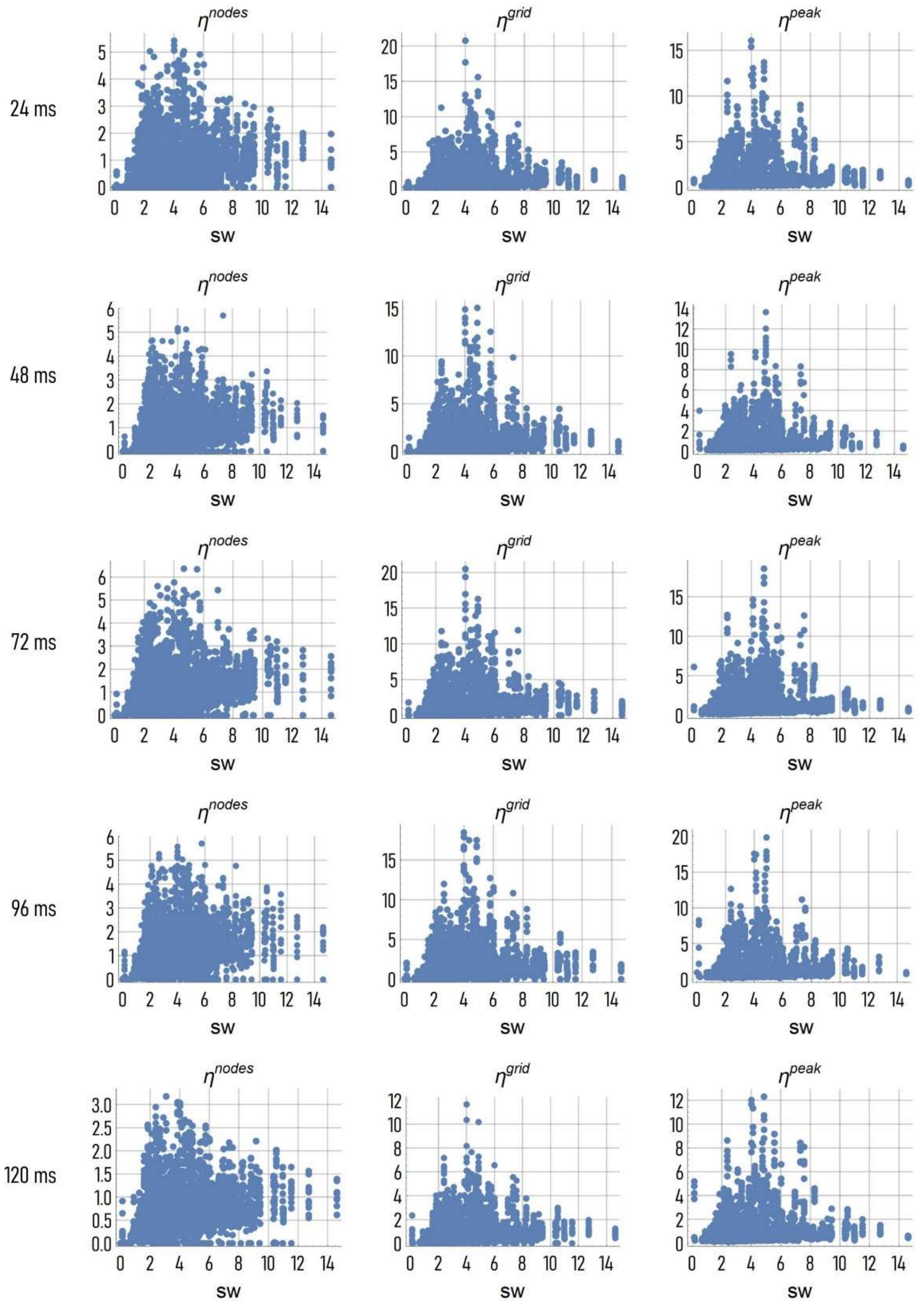

Supplementary Figure 20. Measured values of η^{nodes}, η^{grid} and η^{peak} as a function of the small world coefficient SW, measured for a signal length $\Delta t = 24,\ 48, 72, 96, 120\ ms$, and a frequency $f = 333\ Hz$.

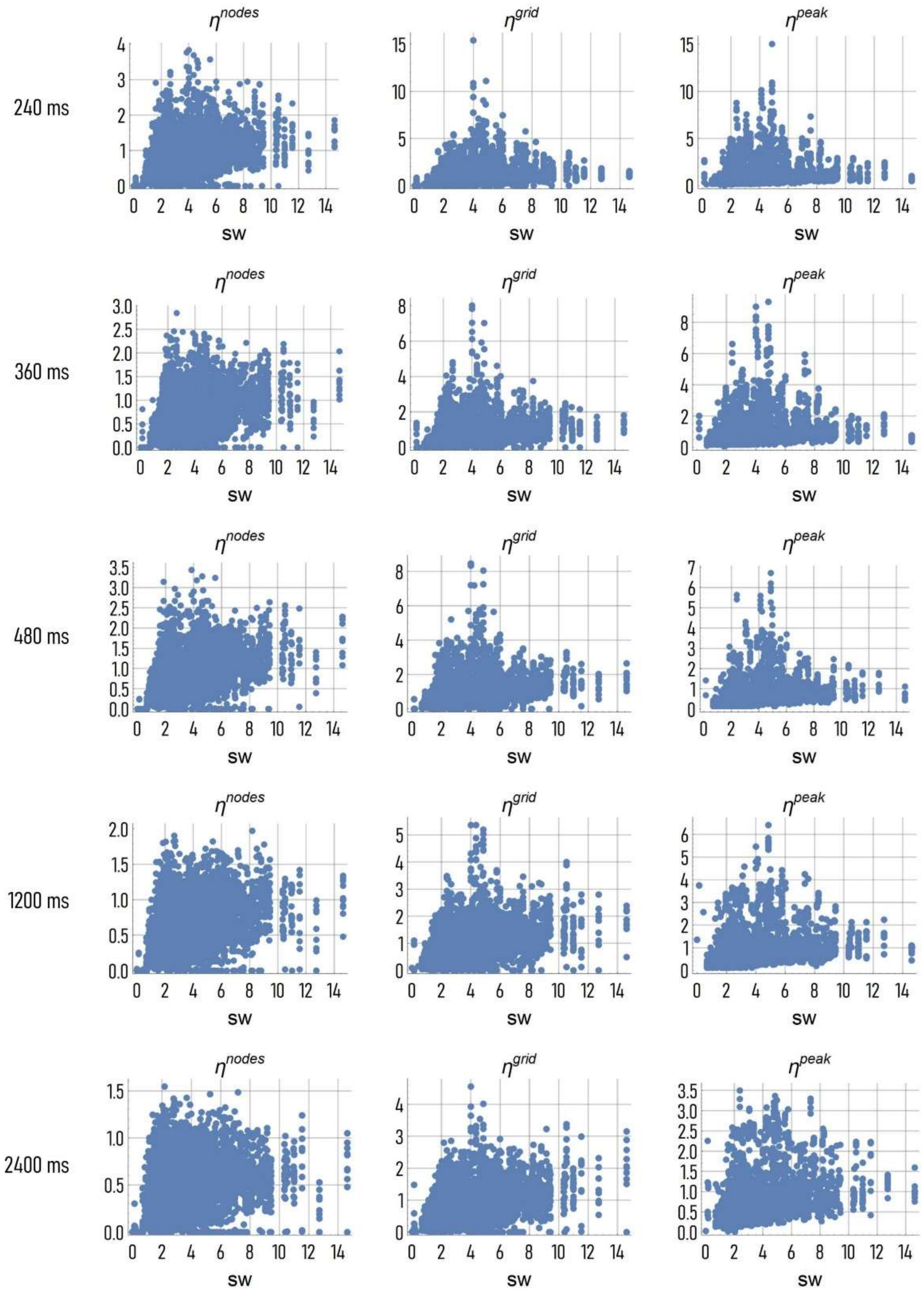

Supplementary Figure 21. Measured values of η^{nodes}, η^{grid} and η^{peak} as a function of the small world coefficient SW, measured for a signal length $\Delta t = 240,\ 360, 480, 1200, 2400\ ms$, and a frequency $f = 333\ Hz$.

Supplementary Information section 3. Values of quality factor associated to η^{grid}, η^{peak} and η^{nodes} calculated for different combinations of signal length (Δt) and signal frequency (f), and for values of the small world coefficient spanning the $1 - 14$ interval.

The quality factor quantifies how the efficiency varies as a function of SW. The quality factor, Q^{x-y}, is the efficiency increment expressed in terms of percentage, evaluated between an initial (x) and final (y) value of small-world-ness. Thus, to make an example, $Q^{1-4} = (\eta_4 - \eta_1)/\eta_1$. The quality factor is non-dimensional, similar to the efficiency.

In the **Supplementary Figure 22a**, we report the grid-efficiency (η^{grid}) as a function of SW for different values of the signal length (Δt), for a fixed frequency of the wave travelling in the networks ($f = 133\ Hz$). The diagram is the completion of the data partially reported in **Figure 3a** of the main text. The quality factor Q^{1-sw} associated to η^{grid} is reported in **Supplementary Figure 22b** as a function of the small-world coefficient of the networks and of Δt. The density-plot illustrates that the maximum enhancement increment is found for intermediate values of SW ($\mathrm{SW} = 4 - 5$) and Δt ($\Delta t = 72 - 240$ ms): for these, Q reaches values as high as $\sim 1200\%$ - indicating a 12 fold increase of efficiency moving from $\mathrm{SW} = 1$ to $\mathrm{SW} = 4 - 5$. To understand how the network efficiency varies for definite values of the small-world-coefficient as a function of Δt, we report in the **Supplementary Figures 22c-f** the quality factor Q^{1-4} (c), Q^{1-5} (d), Q^{14-4} (e), Q^{14-5} (f). These parameters indicate the variation of efficiency in a grid obtained by changing the network topology from $\mathrm{SW} = 1$ and $\mathrm{SW} = 14$ (i.e. values at the extreme of the small-world range) to $\mathrm{SW} = 4$ and $\mathrm{SW} = 5$ (i.e. intermediate values of small-world-ness). For this configuration, the maximum increment is achieved for a final small-world value of $\mathrm{SW} = 4$, for which the quality factor is determined as $Q^{1-4} \sim 1200\%$ (**Supplementary Figure 22c**) and $Q^{14-4} \sim 1000\%$ (**Supplementary Figure 22e**) from an initial layout characterized by $\mathrm{SW} = 1$ and $\mathrm{SW} = 14$, respectively. Conversely, the quality factor is determined as $Q^{1-5} \sim 800\%$ (**Supplementary Figure 22d**) and $Q^{14-5} \sim 780\%$ (**Supplementary Figure 22f**) for a final $\mathrm{SW} = 5$ topology network.

Similarly to the grid-efficiency, the diagram in **Supplementary Figure 23a** illustrates the peak-efficiency (η^{peak}) as a function of SW for different values of the signal length (Δt) for a fixed central frequency $f = 133\ Hz$. For this enhancement factor, the quality factor Q^{1-sw} is reported

in **Supplementary Figure 23b** as a function of the small-world coefficient of the networks and of Δt. In this case, the maximum peak-enhancement increment is determined as $\sim 500\%$ again for intermediate values of small-world-ness, from an initial random configuration (SW $= 1$). Differently from the grid-enhancement case, the maximum quality factor is here obtained for a transformation that brings the network from a topology characterized by SW $= 14$, to a layout with SW $= 4$ ($Q^{14-4} \sim 1000\%$, **Supplementary Figure 23e**) and SW $= 5$ ($Q^{14-5} \sim 1100\%$, **Supplementary Figure 23f**). These values compare to the smaller quality factors $Q^{1-4} \sim 300\%$ (**Supplementary Figure 23c**) and $Q^{1-5} \sim 500\%$ (**Supplementary Figure 23d**).

Diagrams relative to the nodes-efficiency are consistent with the results presented heretofore. The **Supplementary Figure 24a** illustrates the nodes-efficiency (η^{nodes}) as a function of SW for different values of the signal length (Δt) for a fixed central frequency $f = 133\ Hz$. The relative values of quality factor Q^{1-sw} are shown in the **Supplementary Figure 24b**. Coherent with what found before, the nodes-performance is optimized for intermediate values of small-world-ness (SW $= 4 - 5$), for which the quality factor touches values as high as $Q \sim 500\%$. The values of quality factor Q^{1-4}, Q^{1-5}, Q^{14-4}, and Q^{14-5} are reported in the **Supplementary Figure 24c-f**. For these model parameters, the maximum quality factor is determined for a final small-world-ness SW $= 4$, starting from SW $= 1$, for which $Q^{1-4} = 500\%$ for $\Delta t = 96$ ms.

We repeated the same analysis for the data presented in **Figure 4** of the main manuscript. Moving the focus from the signal length (Δt) to the frequency (f) of the travelling disturbance, we have examined how the networks performance varies as a function of SW for different values of f and a fixed $\Delta t = 120$ ms. The evaluation of the networks performance was performed three times: for the grid-efficiency (η^{grid}, **Supplementary Figure 25**), the peak-efficiency (η^{peak}, **Supplementary Figure 26**), and the nodes-efficiency (η^{nodes}, **Supplementary Figure 27**).

Overall, results of the analysis are consistent with the findings reported in the **Supplementary Figures 22-24** for a fixed central frequency and varying value of signal length. The maximum increment of efficiency is found for intermediate values of small-world ness (SW $= 4 - 5$). For certain combinations of the driving frequency and parameters of the model, the quality factors reach values equal to $Q^{1-4} \sim 1250\%$ (η^{grid}), $Q^{1-5} \sim 1000\%$ (η^{peak}) and $Q^{1-5} \sim 320\%$ (η^{nodes}).

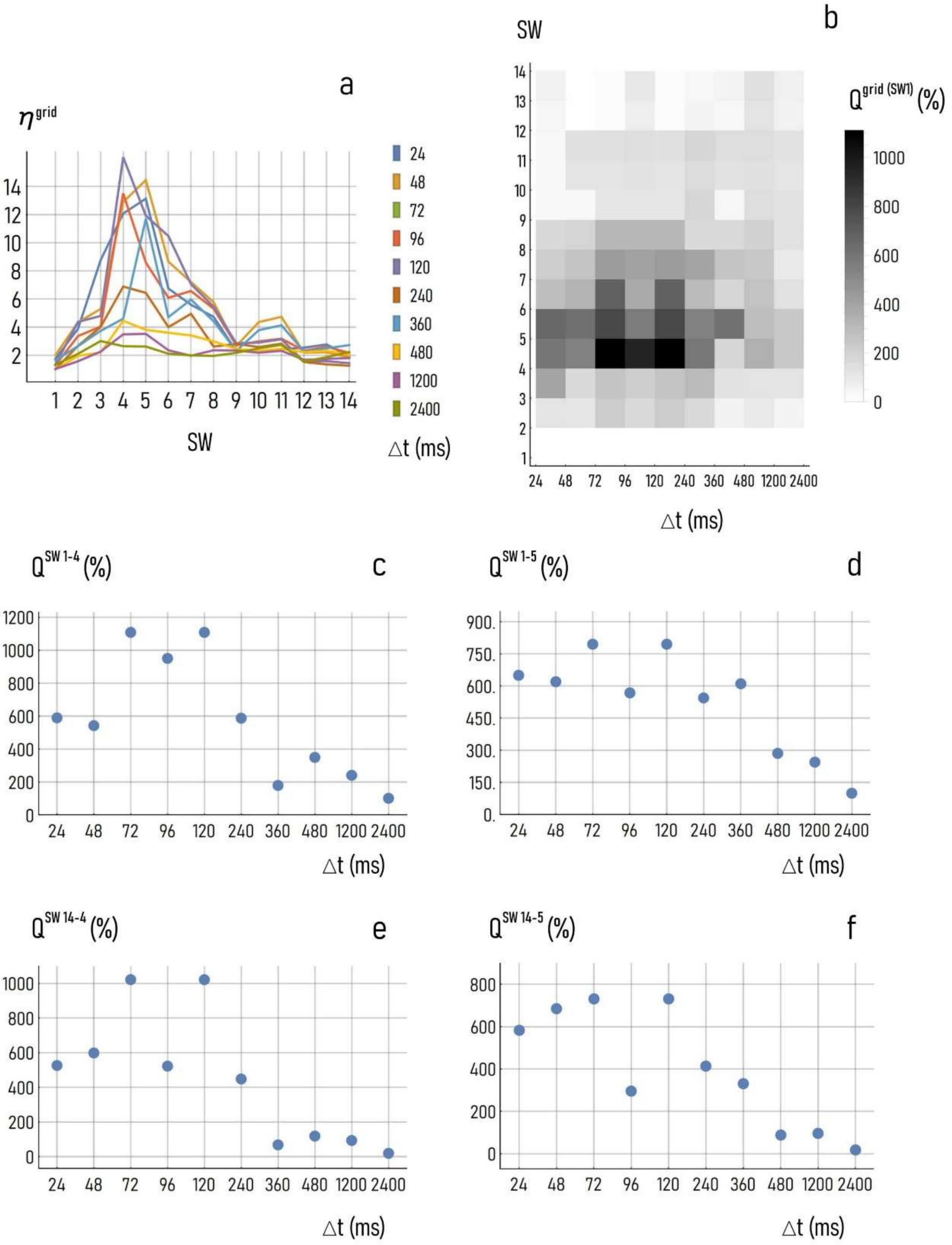

Supplementary Figure 22. Network information efficiency (η^{grid}) as a function of the small-world coefficient for different values of the signal length (a). Quality factor associated to η^{grid} calculated for different values of SW and Δt (b). Quality factor Q^{1-4} (i.e. the normalized relative increment of performance of the grid measured between the values of SW 1 and 4) associated to η^{grid} as a function of Δt (c). Quality factor Q^{14-4} associated to η^{grid} as a function of Δt (d). Quality factor Q^{14-5} associated to η^{grid} as a function of Δt (d).

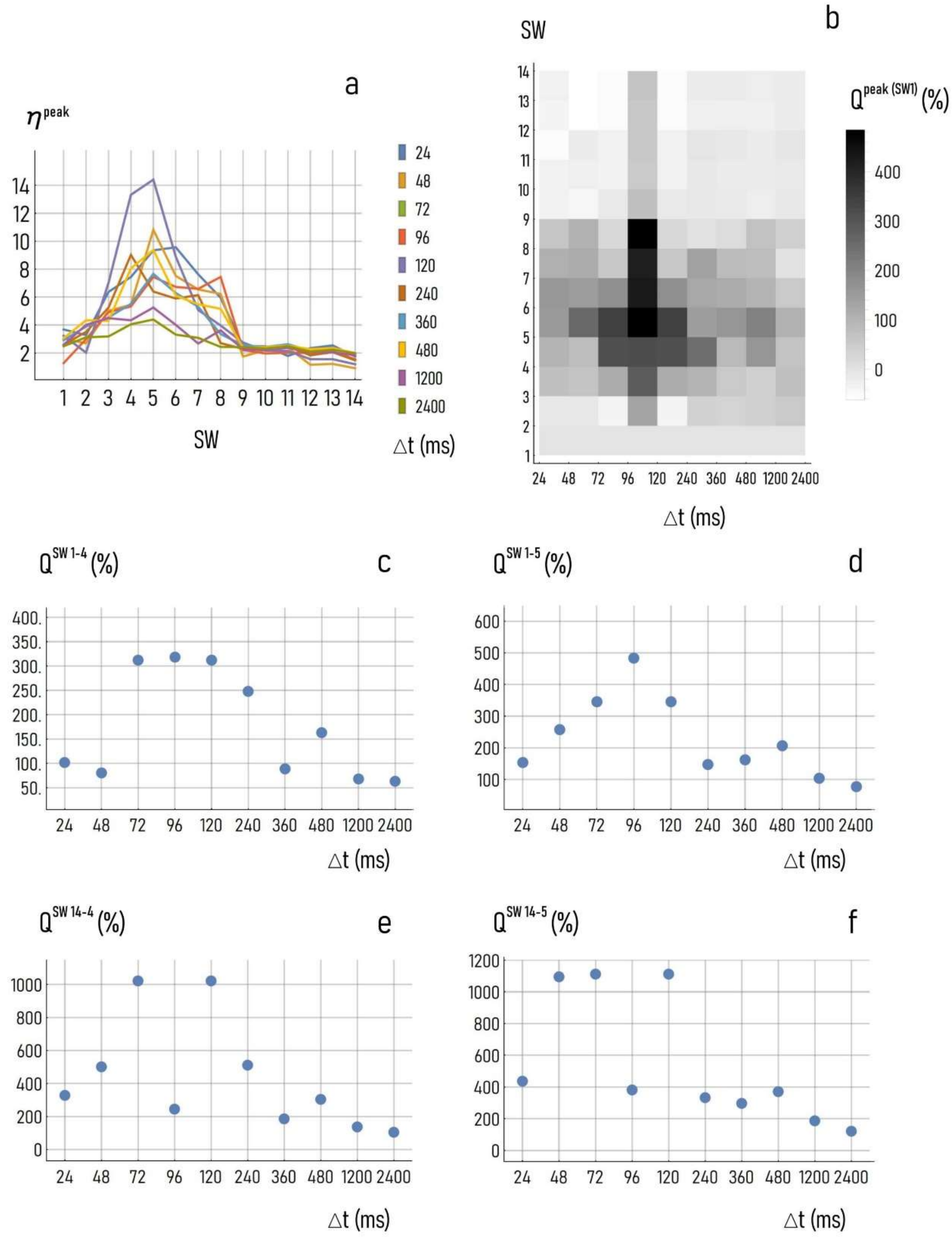

Supplementary Figure 23. Peak information efficiency (η^{peak}) as a function of the small-world coefficient for different values of the signal length (a). Quality factor associated to η^{peak} calculated for different values of SW and Δt (b). Quality factor Q^{1-4} associated to η^{peak} as a function of Δt (c). Quality factor Q^{14-4} associated to η^{peak} as a function of Δt (d). Quality factor Q^{14-5} associated to η^{peak} as a function of Δt (d).

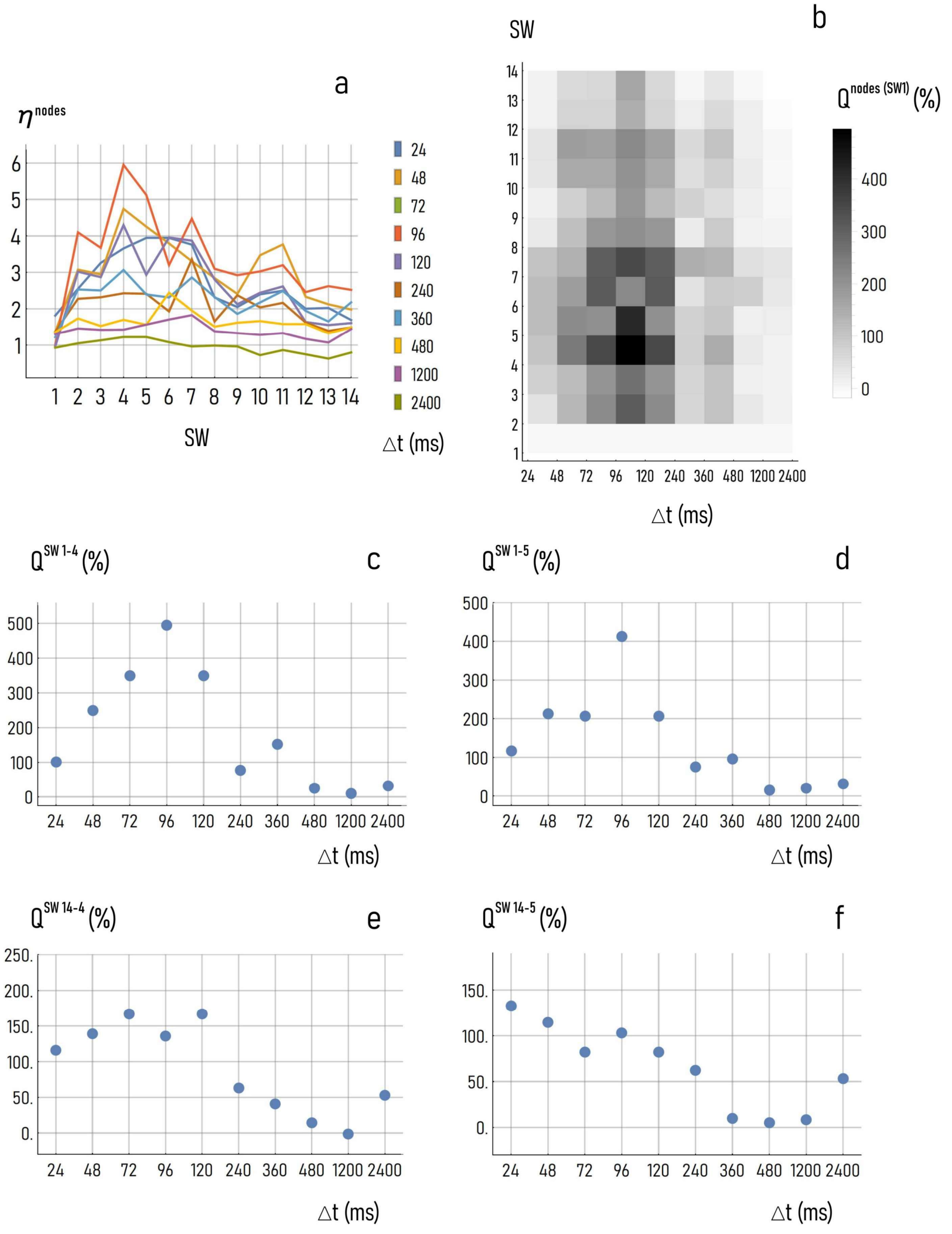

Supplementary Figures 24. Active nodes efficiency (η^{nodes}) as a function of the small-world coefficient for different values of the signal length (a). Quality factor associated to η^{nodes} calculated for different values of SW and Δt (b). Quality factor Q^{1-4} associated to η^{nodes} as a function of Δt (c). Quality factor Q^{14-4} associated to η^{nodes} as a function of Δt (d). Quality factor Q^{14-5} associated to η^{nodes} as a function of Δt (d).

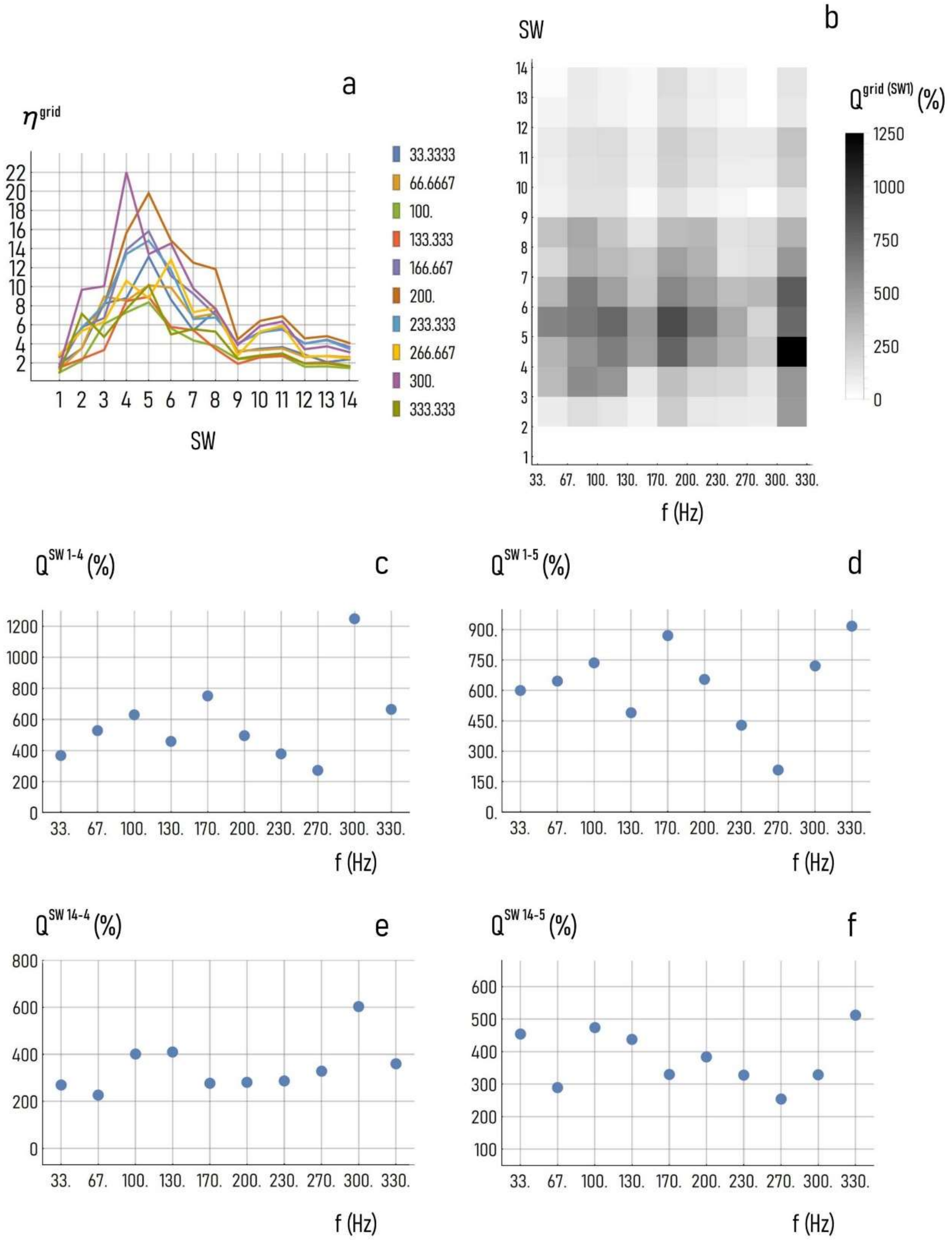

Supplementary Figure 25. Network information efficiency (η^{grid}) as a function of the small-world coefficient for different values of the signal frequency (a). Quality factor associated to η^{grid} calculated for different values of SW and f (b). Quality factor Q^{1-4} associated to η^{grid} as a function of f (c). Quality factor Q^{14-4} associated to η^{grid} as a function of f (d). Quality factor Q^{14-5} associated to η^{grid} as a function of Δt (d).

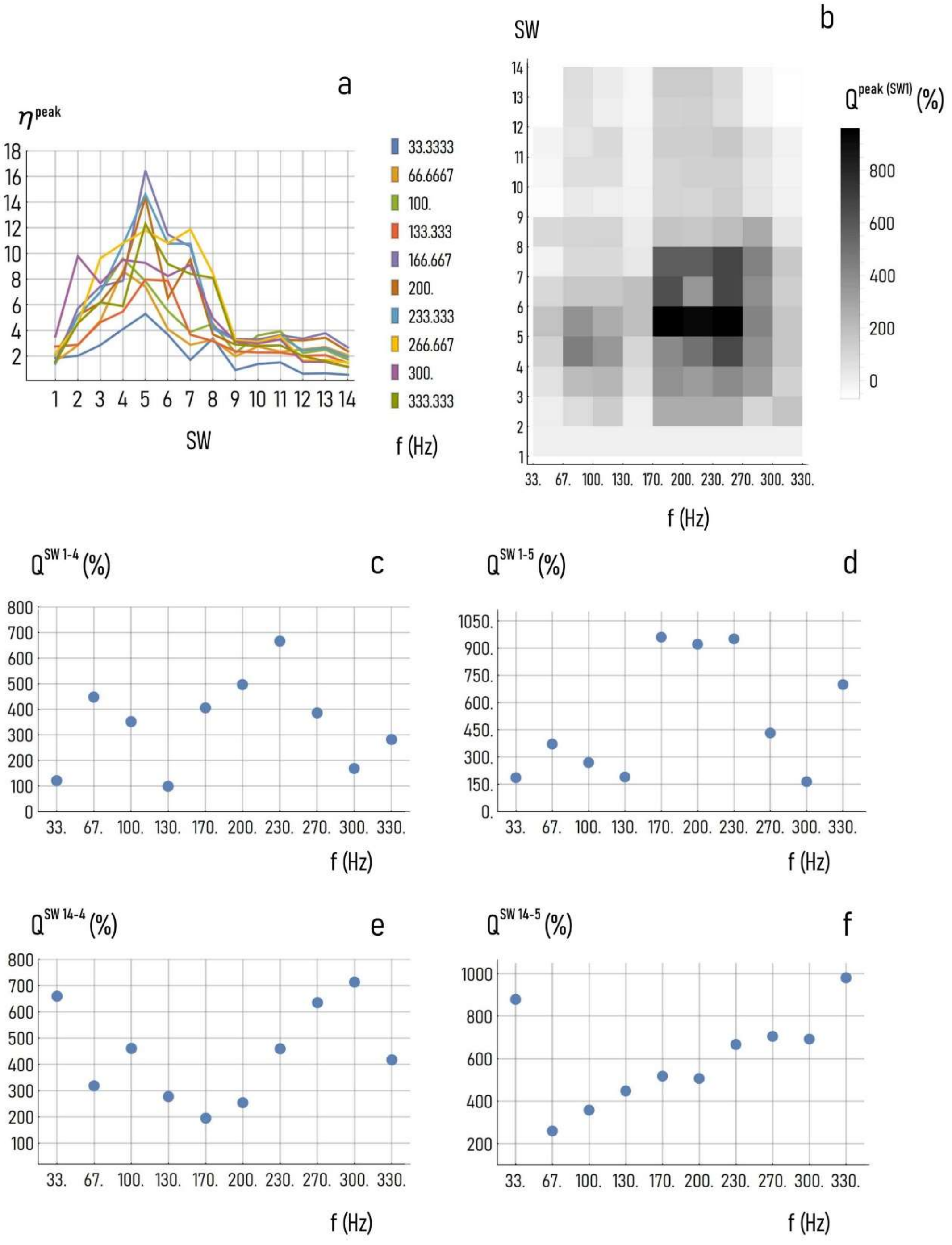

Supplementary Figure 26. Peak information efficiency (η^{peak}) as a function of the small-world coefficient for different values of the signal frequency (a). Quality factor associated to η^{peak} calculated for different values of SW and f (b). Quality factor Q^{1-4} associated to η^{peak} as a function of f (c). Quality factor Q^{14-4} associated to η^{peak} as a function of f (d). Quality factor Q^{14-5} associated to η^{peak} as a function of Δt (d).

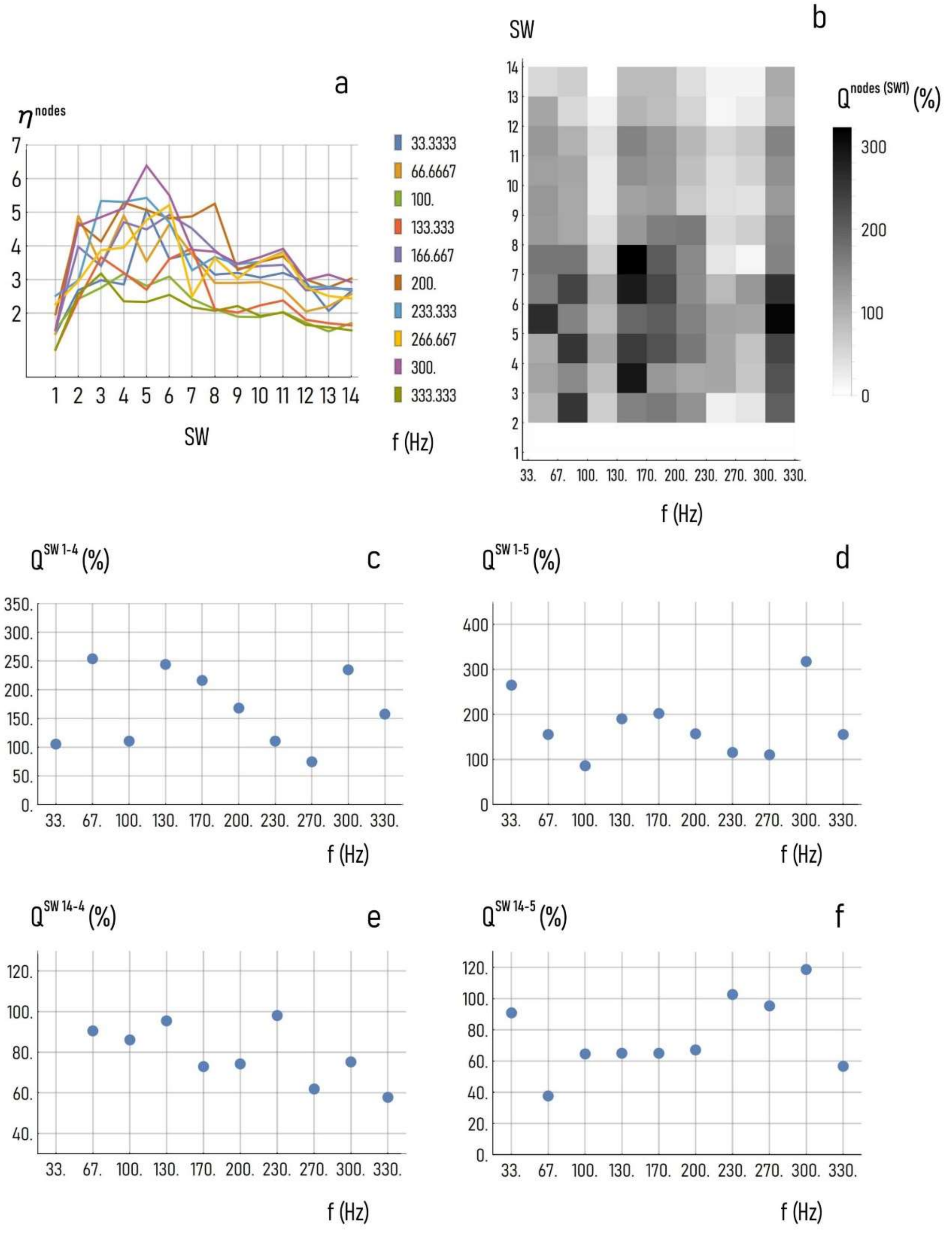

Supplementary Figure 27. Active nodes efficiency (η^{nodes}) as a function of the small-world coefficient for different values of the signal frequency (a). Quality factor associated to η^{nodes} calculated for different values of SW and f (b). Quality factor Q^{1-4} associated to η^{nodes} as a function of f (c). Quality factor Q^{14-4} associated to η^{nodes} as a function of f (d). Quality factor Q^{14-5} associated to η^{nodes} as a function of Δt (d).

Supplementary Information section 4. Network analysis of graphs.

Starting from graphs generated on squared domains having same number of nodes (500) and different layout, we imported the nodes coordinates matrix and the connectivity information (contained in the *adjacency matrix)*, to quantify some network parameters including the clustering coefficient, the characteristic path length and then the 'smallworldness' coefficient. These parameters give an indication of the connectivity properties of the nodes in a graph and allow to distinguish between graphs of different types (regular, random or small world).
In graph theory, the *clustering coefficient* (C_c) is a measure of the degree to which nodes in a graph tend to cluster together. C_c ranges from 0 (none of the possible connections among the nodes are realized) to 1 (all possible connections are realized and nodes group together to form a single aggregate). The *clustering coefficient* is defined as

$$C_i = \frac{2E_i}{k(k-1)} \quad (1)$$

where k is the number of neighbors of a generic node i, E_i is the number of existing connections between those, $k(k-1)/2$ being the maximum number of connections, or combinations, that can exist among k nodes. Notice that the clustering coefficient C_i is defined locally, and a *global* value, C_c, is derived upon averaging C_i over all the nodes that compose the graph.
The *characteristic path length* (Cpl) is defined as the average number of steps along the shortest paths for all possible pairs of network nodes. The *shortest path length* (Spl), between two nodes is the path that connects the two nodes with the shortest number of edges and it is the minimum distance between a generic couple of nodes.
The *characteristic path length* of the networks that we have examined in the work, has been determined by algorithms built-in in the software system Wolfram's Mathematica – that is the software that we have used to generate the many different network configurations where, later, we have verified the transport of information. To the aim of finding the cpl of the networks, we used the function $MeanGraphDistance(G)$, that returns the average length of all shortest paths between vertices of G. Notice that this coincides with the definition of characteristic path length: the average number of edges in the shortest paths between all vertex pairs[1]. The function

$MeanGraphDistance$, in turn, is based on the Dijkstra's algorithm [2], that is a well-assessed method to find the shortest paths between nodes of a graph.

1 Schreiber, F. in *Encyclopedia of Systems Biology* (eds W Dubitzky, O Wolkenhauer, K H Cho, & H Yokota) (Springer, 2013).

2 Dijkstra, E. W. A note on two problems in connexion with graphs. *Numerische Mathematik* **1**, 269–271 (1959).
